# In Situ Polymerized Composite Electrolytes for High‐Performance Solid‐State Lithium Batteries: A Review

**DOI:** 10.1002/advs.75159

**Published:** 2026-04-09

**Authors:** Jialin Li, Yufen Yan, Xiaoju Wang, Yuhui Huang, Zhen Huang, Lingjie Zhang, Ningzhong Bao, Zijian Hong, Yongjun Wu, Dali Ji

**Affiliations:** ^1^ Zhejiang Key Laboratory of Advanced Solid State Energy Storage Technology and Applications Taizhou Institute of Zhejiang University Taizhou China; ^2^ State Key Laboratory of Silicon and Advanced Semiconductor Materials School of Materials Science and Engineering Zhejiang University Hangzhou Zhejiang China; ^3^ Institute of Fundamental and Transdisciplinary Research Zhejiang University Hangzhou China; ^4^ School of Engineering Hangzhou City University Hangzhou Zhejiang China

**Keywords:** composite solid electrolytes, In‐situ polymerization, interfacial compatibility, solid‐state lithium‐ion battery

## Abstract

Solid‐state electrolytes offer improved safety compared to liquid counterparts but suffer from inherent limitations when used as single‐component systems. Composite electrolytes, combining complementary materials, provide a promising route to overcome these issues. Among fabrication techniques, in situ polymerization stands out for its ability to construct intimately integrated composite polymer electrolytes with enhanced interfacial stability and tunable architectures. However, a systematic and comprehensive review of recent advances in in situ polymerized composite polymer electrolytes (CPEs) is still lacking. In this work, recent developments in in situ polymerized CPEs are systematically reviewed and categorized based on different polymer matrices and their specific interactions with various inorganic fillers. The fundamental reaction mechanisms governing in situ polymerization are discussed, with particular attention to the coupling between polymerization kinetics, interfacial structure evolution, and ion‐transport pathway reconstruction. Building on this mechanistic perspective, representative polymer–filler systems are analyzed to illustrate how organic–inorganic interactions regulate ionic conductivity, mechanical integrity, and electrochemical stability. Key challenges in current systems are critically assessed, and finally, future perspectives, guidelines, and strategies toward the resolution of these challenges are analyzed and discussed.

## Graphical abstract

This review systematically explores the recent advances in in situ polymerized composite polymer electrolytes (CPEs) for solid‐state lithium batteries. It covers the fundamentals of reaction mechanisms, monomer chemistry, and their impact on interfacial stability, ionic conductivity, and electrochemical performance. The review also highlights the challenges and future perspectives on optimizing CPEs for practical applications.

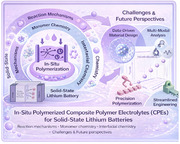

## 1 Introduction

With the accelerating challenges posed by global climate change and growing concerns over energy security, the development of advanced electrochemical energy storage systems has become a critical area of research. Lithium‐ion batteries (LIBs) have emerged as one of the most promising solutions due to their high energy density, long cycle life, and exceptional power performance [1]. LIBs are typically composed of four main components: the cathode, anode, electrolyte, and separator. The electrochemical reactions occurring at the cathode and anode determine the battery's voltage and capacity, while the electrolyte serves as a medium for transporting lithium ions between these two electrodes. The separator is placed between the cathode and anode to prevent internal short circuits while still allowing lithium ion transport. In conventional LIBs, liquid organic electrolytes like ethylene carbonate are commonly used because they demonstrate good ionic conductivity and high stability [2]. However, these liquid electrolytes are flammable, presenting significant safety concerns during thermal runaway [3]. Solid‐state lithium‐ion batteries (SSLIBs) address these safety issues by replacing flammable liquid electrolytes with solid‐state electrolytes (SSEs). This change offers substantial advantages in safety, electrochemical stability, and the potential for higher energy density when paired with lithium metal anodes.

SSEs can be typically classified into three categories: inorganic solid electrolytes, polymer electrolytes, and composite solid polymer electrolytes (CPEs). Inorganic solid electrolytes, which include oxides [4, 5, 6, 7], sulfides [8, 9, 10], and halides [11, 12, 13, 14], are known for their high ionic conductivity, wide electrochemical stability windows, excellent thermal stability, and high mechanical modulus which could effectively suppress lithium dendrite growth. However, their inherent brittleness, poor processability, and high interfacial resistance limit their practical application in solid‐state batteries. In contrast, polymer electrolytes offer superior flexibility, good film‐forming ability, and excellent interfacial compatibility with electrodes, alongside ease of processing and improved safety. Nonetheless, their low room‐temperature ionic conductivity and limited compatibility with high‐voltage cathodes remain significant challenges. CPEs have emerged as a promising alternative by incorporating inorganic fillers (high ionic conductivity and structural stability) into polymer matrices (high mechanical flexibility and good interfacial contact). Through careful tuning of polymer matrices, filler types, and microstructures, CPEs can achieve synergistic enhancements in mechanical, interfacial, and electrochemical properties, positioning them as promising candidates for next‐generation solid‐state lithium batteries [15, 16, 17, 18].

To date, various synthesis techniques for CPEs have been developed, such as solution casting [19, 20], melt‐quenching [21, 22], vapor deposition [23, 24], atom layer deposition [25, 26, 27], and in situ polymerization [28, 29, 30, 31, 32, 33, 34, 35, 36]. Among them, in situ polymerization has attracted increasing attention as an emerging and versatile strategy for fabricating composite polymer electrolytes [37, 38, 39, 40] This method involves mixing monomers or oligomers with inorganic fillers, followed by polymerization directly within the composite system. Such an approach enables the formation of intimate interfacial contact between the polymer matrix and the inorganic phase, leading to significant improvements in ionic conductivity, mechanical integrity, and interfacial stability. To clarify the distinctions between in situ polymerization and other commonly employed fabrication techniques for composite polymer electrolytes, Table 1 summarizes representative synthesis strategies together with their processing characteristics, structural features, and typical advantages and limitations.

**TABLE 1:** Comparison of representative synthesis strategies for CPEs.

Fabrication strategy	Processing principle	Structural characteristics	Advantages	Limitations	References
Solution casting	Polymer and fillers dissolved/dispersed in solvent, followed by solvent evaporation to form film	Polymer matrix formed after solvent removal; fillers physically dispersed	Simple processing; good film uniformity	Residual solvent; limited interfacial contact with electrodes	[19, 20]
Melt‐quenching	Polymer melted and mixed with fillers, then rapidly cooled to form solid electrolyte	Dense polymer matrix; fillers embedded in molten polymer	Solvent‐free; scalable	High processing temperature; limited compatibility with electrodes	[21, 22]
Vapor deposition	Polymer or inorganic components deposited from vapor phase onto substrate	Thin and uniform coating layers	Precise thickness control; conformal coating	Complex equipment; difficult for bulk electrolyte fabrication	[23, 24]
Atomic layer deposition	Sequential self‐limiting surface reactions forming ultrathin layers	Atomic‐level control of interfacial layers	Excellent interface engineering capability	Extremely slow deposition; mainly used for interface modification	[25, 26]
in situ polymerization	Monomers mixed with salts and fillers, then polymerized directly inside the composite or cell	Polymer network formed around fillers and electrodes; reaction‐constructed interfaces	Conformal interfacial contact; improved ion transport continuity; reduced interfacial resistance	Polymerization kinetics must be carefully controlled	[29, 31, 33]

While numerous studies have reported in situ polymerized CPEs with improved electrochemical performance, a mechanistic understanding of how polymerization reactions govern structural evolution and interfacial integration remains insufficient. Unlike conventional solution‐cast systems, in situ polymerization involves simultaneous monomer conversion, network formation, and interface construction within the battery architecture. As a result, polymerization kinetics, crosslinking behavior, filler surface chemistry, and ion transport pathways become intrinsically coupled rather than independently optimized parameters.

This intrinsic reaction–structure–interface coupling raises several fundamental questions: How does polymerization rate influence interfacial wetting and stress development? To what extent do reactive intermediates interact with inorganic fillers, particularly active solid electrolytes? How does monomer chemistry dictate the formation of ion‐conducting networks across polymer and inorganic phases? Addressing these questions requires a reaction‐governed perspective that connects molecular design with interfacial evolution and electrochemical functionality.

**FIGURE 1:**
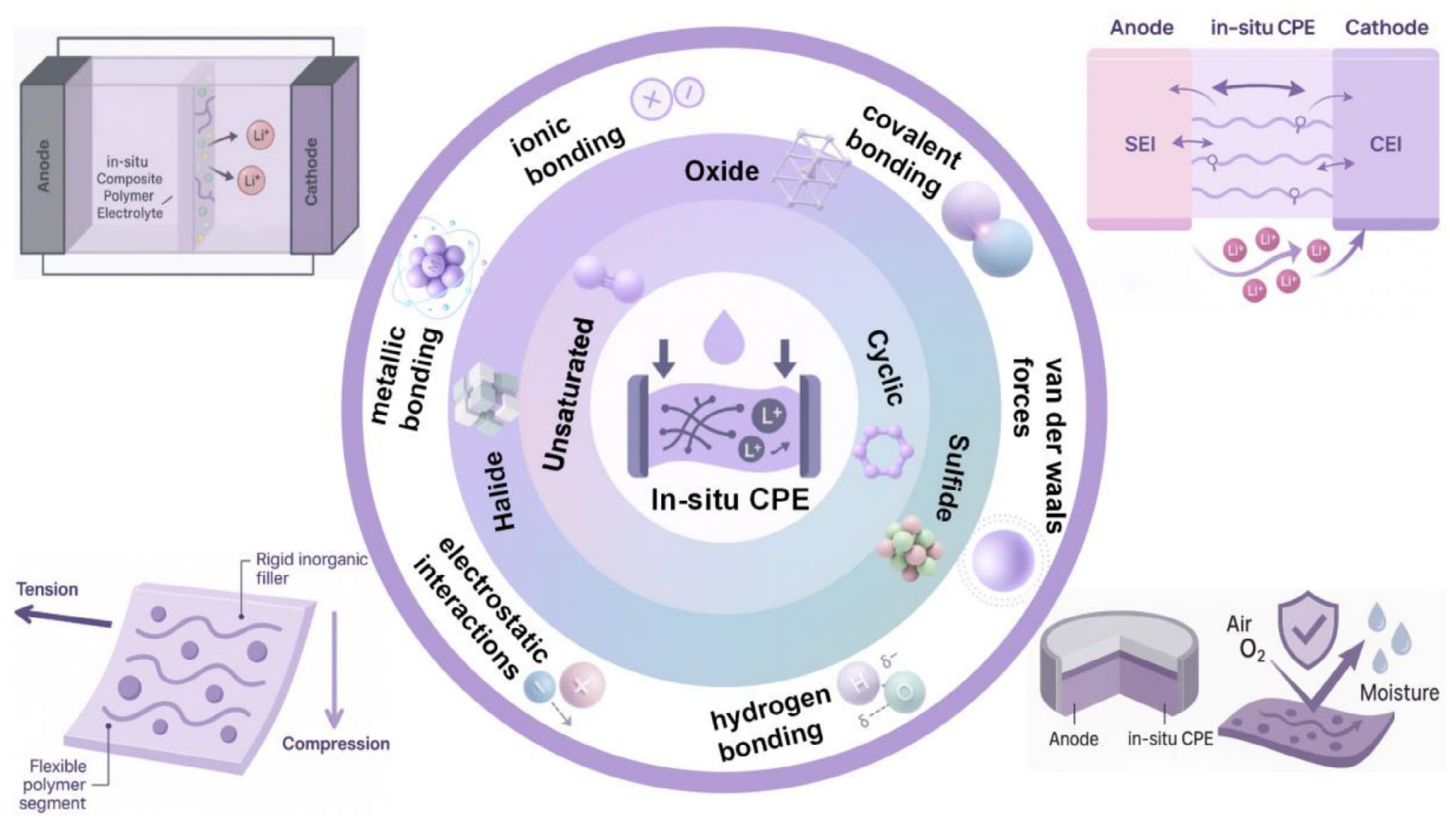
Correlation between structure and performance of in situ polymerized composite electrolytes.

In this review, we systematically analyze in situ polymerized composite polymer electrolytes from the standpoint of monomer chemistry, recognizing that the molecular structure of polymerizable units fundamentally determines polymerization pathways, network architecture, dielectric properties, and interfacial compatibility. Based on this principle, CPEs systems are classified according to the type of polymerizable monomer units, including unsaturated monomers and cyclic monomers. Within each category, the interactions between the evolving polymer matrix and inorganic fillers (include oxides, sulfides and halides)—are critically examined, with particular emphasis on reaction kinetics, interfacial coupling mechanisms, and ion transport reconstruction, as illustrated in Figure 1 [41, 42].

By establishing correlations among reaction systems, structural features, and electrochemical behavior, this review aims to clarify the mechanistic foundation of in situ polymerized CPEs and to provide design principles for next‐generation solid‐state lithium batteries. We further identify the key challenges that still hinder practical implementation and highlight that progress will depend on establishing robust, quantitative links between reaction conditions, heterogeneous microstructures, and cell‐level behaviors under realistic operating constraints. Lastly, future research directions are presented, including the integration of emerging intelligent design strategies, data‐driven discovery with precision polymerization control, operando‐enabled interfacial diagnosis, and scalable process engineering to accelerate the development of next‐generation in situ polymerized CPEs.

## 2 Reaction Principles and Interfacial Evolution in in situ Polymerized Composite Electrolytes

In situ polymerization in composite polymer electrolytes is not merely a processing strategy, but a reaction‐driven structural construction process occurring within the electrochemical cell environment. Unlike ex situ fabricated systems, where polymer films are prepared independently and subsequently assembled with electrodes, in situ systems undergo polymer chain growth, network formation, and interfacial integration simultaneously in the presence of inorganic fillers and electrode surfaces. Consequently, chemical reactions, mechanical evolution, and interfacial stabilization are inherently interdependent rather than sequentially controlled.

This simultaneity fundamentally alters the formation mechanism of composite electrolytes. Polymerization kinetics govern not only molecular weight growth and crosslink density, but also monomer infiltration into porous electrodes, local viscosity evolution, and stress development during curing. At the same time, inorganic fillers—particularly ionically conductive solid electrolytes—may influence radical stability, surface reactions, and local ion redistribution, thereby coupling reaction pathways with interfacial chemistry. The resulting microstructure is therefore a product of dynamic reaction–interface interactions rather than simple phase blending.

Importantly, this reaction‐driven construction introduces an intrinsic design contradiction: rapid polymerization and high crosslink density enhance mechanical integrity and interfacial fixation, yet may simultaneously restrict segmental mobility, limit monomer penetration, and increase internal stress; similarly, highly reactive intermediates can promote interfacial bonding while potentially destabilizing chemically sensitive inorganic phases. Balancing these competing effects is central to the rational design of in situ polymerized CPEs.

To establish such balance, it is essential to adopt a reaction‐governed framework that connects polymerization mechanisms with structural evolution and ion transport reconstruction. In this section, we discuss the fundamental reaction kinetics, network formation characteristics, interfacial transformation processes, and filler‐dependent effects that collectively determine the architecture and electrochemical behavior of in situ composite electrolytes.

### 2.1 Polymerization Kinetics and Initiation Pathways

Polymerization in in situ composite polymer electrolytes is fundamentally governed by the kinetics of initiation and propagation reactions occurring within a heterogeneous electrochemical environment. Unlike conventional bulk polymerization, where monomers react in relatively homogeneous media, in situ systems undergo polymer chain growth in the presence of electrode surfaces and inorganic fillers. Consequently, the stability of reactive intermediates, the diffusion of monomers, and the local reaction rate are all strongly influenced by interfacial interactions and spatial confinement. The resulting polymer network therefore reflects not only intrinsic reaction chemistry but also the local physicochemical conditions within the composite system.

Several initiation pathways are commonly employed in in situ polymerized electrolytes, including free‐radical polymerization, cationic polymerization, and ring‐opening polymerization [43]. Free‐radical polymerization is particularly prevalent in acrylate‐based systems due to its relatively mild reaction conditions and compatibility with electrochemical components [44]. In these systems, thermal or photochemical initiators such as azobisisobutyronitrile (AIBN) or benzoyl peroxide generate radical species that initiate chain growth through successive monomer addition [45]. The rate of polymerization is therefore determined by the balance between radical generation, propagation, and termination processes, which collectively dictate the evolution of molecular weight and network formation.

Representative experimental systems illustrate how these initiation mechanisms directly influence electrochemical performance. Acrylate‐based monomers such as polyethylene glycol diacrylate (PEGDA) have been widely used to construct crosslinked polymer networks through free‐radical polymerization. In PEGDA‐based in situ gel electrolytes, ionic conductivities exceeding 1.2 mS cm^−1^ at 25°C and electrochemical stability windows approaching 4.6 V vs. Li^+^/Li have been reported, demonstrating the ability of radical polymerization systems to form highly conductive polymer networks under relatively mild processing conditions [46]. Similarly, crosslinked electrolytes based on pentaerythritol tetracrylate (PETEA) initiated by AIBN have shown ionic conductivities of approximately 1.03 mS cm^−1^at room temperature, lithium‐ion transference numbers of 0.73, highlighting the ability of multifunctional monomers to produce mechanically robust yet ionically conductive networks [47].

In contrast to radical polymerization, certain in situ electrolytes rely on ring‐opening polymerization of cyclic ethers [48]. Monomers such as dioxolane (DOL) or polyethylene glycol diglycidyl ether (PEGDE) can undergo cationic or anionic ring‐opening reactions initiated by lithium salts or Lewis‐acid catalysts [49, 50]. These reactions produce polymer chains with flexible ether linkages that are intrinsically favorable for Li^+^ coordination and transport. In PEGDE‐based systems incorporating catalytic inorganic frameworks such as aluminum alkoxide nanowires, ionic conductivities as high as 7.7 × 10^−4^ S cm^−^^1^ at 30°C have been achieved, illustrating the strong coupling between polymerization pathways and filler‐mediated ion transport [51] To facilitate a clearer comparison of these polymerization pathways, Table 2 summarizes representative in situ polymerization mechanisms together with their processing conditions, network formation characteristics, and electrochemical performance. By juxtaposing radical polymerization systems based on unsaturated monomers with ring‐opening polymerization of cyclic precursors, the table highlights how reaction mechanisms ultimately dictate interfacial structure and ion transport properties.

**TABLE 2:** Reaction–Kinetics–Interface–Transport comparison of representative in situ polymerization systems for CPEs.

Polymerization mechanism	Representative systems	Initiation conditions	Key kinetic feature	Network formation characteristics	Ionic conductivity (typical)	Electrochemical stability window	References
Free‐radical polymerization	MMA, PEGMA	Thermal initiators (AIBN, BPO) 60–80°C or UV photoinitiation	Chain‐growth polymerization; prone to auto‐acceleration (Trommsdorff effect) and vitrification at high conversion	Rapid crosslink formation; tunable network density but possible curing shrinkage	10^−5–^10^−4^ S cm^−1^	∼4.2–4.5 V	[52, 53, 54, 55]
Ring‐opening polymerization (ROP)	DOL, caprolactone	Cationic or Lewis‐acid initiators; typically 25°C–80°C	Stepwise propagation; lower volumetric shrinkage and more uniform network evolution	Flexible polymer chains; lower crosslink density but improved conformal wetting of electrode pores	10^−4–^10^−3^ S cm^−1^	∼4.0–4.5 V	[54, 56]
Cationic polymerization	Epoxy resins, vinyl ether monomers	Lewis acid catalysts or photo‐acid generators	Fast propagation with delayed termination; relatively insensitive to oxygen inhibition	Dense crosslinked networks with strong interfacial adhesion	10^−5–^10^−4^ S cm^−1^	up to ∼4.5 V	[57, 58]
Anionic polymerization	Cyanoacrylates,	Strong nucleophiles or basic initiators	Highly sensitive to impurities; rapid initiation at active surfaces	Fast network formation but limited process control in complex electrode structures	10^−5–^10^−4^ S cm^−1^	∼4 V	[59, 60]

Beyond initiating polymer chain growth, reactive intermediates generated during polymerization may also interact directly with inorganic solid electrolytes embedded within the composite structure. Radical species produced from thermal initiators possess high chemical reactivity and can interact with surface defect sites or chemically labile structural units of inorganic phases. In sulfide electrolytes, for example, radical exposure may perturb P─S bonding environments at the interface [61], potentially inducing subtle surface reconstruction or localized decomposition. In oxide electrolytes such as LLZO or LATP, surface hydroxyl groups and Lewis acidic metal centers may participate in side reactions with monomers or initiator fragments, thereby modifying local surface chemistry [62, 63]. Although these reactions are typically confined to nanometer‐scale interfacial regions, they may significantly influence the spatial distribution of polymerization kinetics and interfacial stability. Polymerization in composite electrolytes should therefore be regarded not as a purely homogeneous chemical reaction but rather as a reaction–interface coupled process, in which radical lifetimes, monomer diffusion, and surface chemistry collectively determine the architecture of the resulting polymer network.

### 2.2 Crosslinking, Molecular Weight Evolution, and Volume Shrinkage

Following initiation, the evolution of polymer molecular weight and crosslink density determines the structural architecture of the polymer electrolyte network. In in situ polymerized composite systems, multifunctional monomers or crosslinking agents frequently generate 3D polymer networks whose connectivity evolves continuously during the curing process [64]. This network formation enhances mechanical stability and enables intimate interfacial integration with electrode surfaces, but it simultaneously alters the local transport environment for lithium ions.

As polymer chains grow and crosslinking reactions proceed, the viscosity of the reaction medium increases significantly. This progressive viscosity rise restricts the mobility of monomers and reactive intermediates, thereby slowing the propagation step and potentially trapping unreacted species within the forming network. In highly crosslinked systems, the polymerization process may approach a vitrification regime in which the glass transition temperature of the polymer approaches the reaction temperature, effectively arresting further molecular rearrangement. The resulting polymer network therefore contains a distribution of chain lengths, crosslink densities, and local free‐volume environments [65].

Experimental studies have demonstrated that the balance between crosslink density and polymer segmental mobility strongly influences ion transport properties [66]. For instance, crosslinked PEG‐based gel polymer electrolytes formed through in situ polymerization have exhibited ionic conductivities in the range of 1.2 to 2.1 × 10^−4^ S cm^−1^ at 60°C [67], depending on the degree of crosslinking and solvent incorporation. Increasing crosslink density enhances mechanical strength and suppresses dendrite penetration, yet excessive crosslinking restricts polymer segmental motion and raises the activation energy for Li^+^ migration.

Network formation is also accompanied by polymerization‐induced volume shrinkage, a phenomenon arising from the conversion of monomer units into covalently bonded polymer chains with shorter intermolecular distances. In confined battery environments, this shrinkage can generate internal stresses at electrode–electrolyte interfaces. If not properly accommodated, these stresses may lead to microscopic gaps or local delamination, thereby interrupting ionic conduction pathways.

The incorporation of inorganic fillers further modifies this structural evolution. Nanoparticles or solid‐state electrolyte particles can serve as heterogeneous nucleation sites that alter polymer chain growth and crosslink distribution. In composite electrolytes containing SiO_2_ nanoparticles, Lewis acid–base interactions between surface Si‐O groups and polymer chains can reduce crystallinity and increase polymer segmental mobility.

These observations highlight that network formation in in situ polymerized composite electrolytes is governed by a complex interplay between polymerization kinetics, crosslink density evolution, and filler‐polymer interactions. The resulting microstructure ultimately determines not only mechanical robustness but also the continuity of Li^+^ transport pathways and the stability of electrode–electrolyte interfaces.

### 2.3 Interfacial Structure Evolution During Polymerization

The formation of polymer network structures in in situ polymerized composite electrolytes provides the structural foundation for the electrolyte matrix; however, once network connectivity and crosslink distribution progressively stabilize during curing, the dominant factor governing electrochemical performance shifts from bulk structural evolution to interfacial functionality [68, 69]. At this stage, the electrode–electrolyte boundary becomes the primary kinetic and chemical constraint controlling lithium‐ion transport, charge transfer continuity, and long‐term cycling stability. Therefore, interfacial regulation represents the most critical design target after network formation is established.

The interfacial evolution described in the preceding sections does not occur independently of electrode architectures or filler surfaces. As polymer chain growth and crosslinking reactions proceed, the electrolyte network simultaneously develops along solid boundaries, giving rise to a reaction‐constructed interphase rather than a simple physical interface [70]. In contrast to ex‐situ polymer electrolytes, where interfacial contact is established only after electrolyte fabrication through mechanical lamination, in situ polymerization enables monomer precursors to infiltrate porous electrodes and filler assemblies prior to network solidification. Consequently, the polymer phase is formed directly within the electrochemically active architecture, promoting conformal interfacial contact and suppressing the initial formation of charge‐transport‐disrupting voids [71].

The early stage of interfacial formation is dominated by monomer infiltration and surface wetting dynamics [72]. Because precursor solutions typically exhibit low viscosity, monomer species can penetrate the porous framework of composite cathodes and distribute around active particles, conductive additives, and inorganic electrolyte domains. This infiltration process establishes a precursor contact configuration that minimizes interfacial heterogeneity before polymerization‐induced immobilization occurs [73]. As polymerization progresses and network viscosity increases, the evolving polymer matrix kinetically stabilizes the conformal geometry established during infiltration, thereby preserving continuous lithium‐ion transport channels across electrode–electrolyte boundaries.

Beyond geometric wetting effects, chemical coupling between polymer segments and inorganic surfaces plays a decisive role in stabilizing the soft–hard interface [74]. Polymer chains bearing polar electron‐donating functional groups, such as ether oxygen, carbonyl, or nitrile moieties, can coordinate with surface lithium species or unsaturated metal coordination centers exposed on inorganic fillers and electrode surfaces [75]. These coordination or dipole‐mediated interactions generate partially immobilized interfacial polymer domains in which segmental mobility is locally suppressed. Such interfacial anchoring enhances adhesion between the mechanically compliant polymer matrix and rigid inorganic phases, facilitating stress transfer across heterogeneous interfaces and reducing the probability of interfacial slippage during repeated electrochemical cycling [76].

**FIGURE 2:**
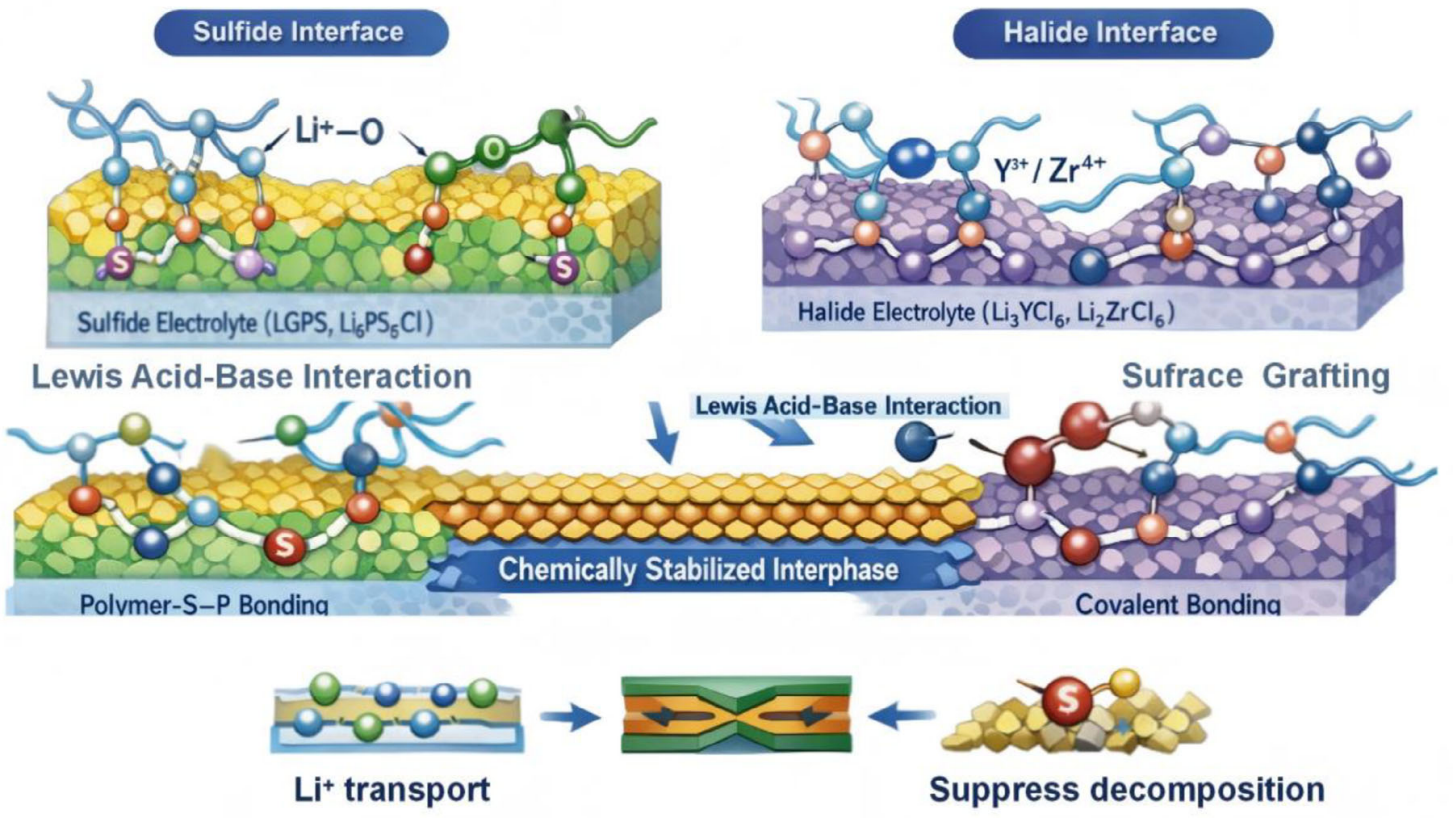
Reaction‐constructed interfacial stabilization mechanisms in in situ CPEs.

This chemical integration is particularly important for composite electrolytes incorporating chemically sensitive solid conductors such as sulfide‐ and halide‐based electrolytes [77, 78]. As shown in Figure 2, the stabilization of the polymer–inorganic interface in in situ polymerized composite electrolytes can arise from multiple complementary interaction mechanisms that depend on the chemical characteristics of the inorganic phase. For sulfide‐based solid electrolytes, such as LGPS, the interfacial coupling is primarily mediated by lithium‐ion coordination and Lewis acid–base interactions. The highly polarizable sulfide framework exposes lithium sites that can interact with electron‐donating functional groups along polymer chains, particularly ether oxygen atoms or other polar moieties. Through these Li─O coordination interactions, polymer segments become partially anchored to the sulfide surface, forming a stabilized interfacial region where the polymer network conforms to the topography of the inorganic framework. Simultaneously, sulfur species within the sulfide lattice can participate in Lewis acid–base interactions with polar groups on the polymer backbone, further strengthening interfacial adhesion and promoting the formation of a chemically integrated soft–hard interface [78, 79, 80]. In contrast, halide solid electrolytes, such as Li_3_YCl_6_ or Li_2_ZrCl_6_, tend to stabilize polymer interfaces through metal‐centered coordination interactions and surface grafting processes. The exposed metal cations at halide surfaces provide coordination centers capable of interacting with electron‐rich functional groups on polymer chains. These interactions facilitate the formation of anchored polymer segments directly bound to the inorganic surface. In some cases, reactive surface sites can further initiate surface‐mediated polymer grafting, enabling polymer chains to propagate from the inorganic interface and generating covalently or coordinatively tethered interfacial layers. Compared with simple physical contact, such chemically anchored interphases exhibit improved structural integrity and resistance to interfacial degradation.

As these interfacial interactions develop, a chemically stabilized interphase emerges at the boundary between the polymer matrix and the inorganic electrolyte particles. Within this transition region, polymer segments are partially immobilized through coordination bonding or surface grafting, while still maintaining sufficient segmental flexibility to support lithium‐ion transport. This hybrid interfacial architecture effectively bridges the mechanical mismatch between the compliant polymer phase and the rigid inorganic particles. The formation of this reaction‐constructed interphase produces several beneficial electrochemical consequences, as summarized in the lower part of Figure 2 [81, 82].

Mechanical evolution accompanying polymerization also contributes to interfacial stabilization. Network formation is inherently associated with volumetric contraction resulting from structural transformation from weakly associated monomer assemblies to covalently bonded polymer frameworks [83]. In radical polymerization systems, curing shrinkage is typically within approximately 3%–10%. Within the spatially confined environment of battery assemblies, excessive shrinkage may generate interfacial tensile stress and promote localized delamination or microvoid nucleation. Therefore, polymerization kinetics should be carefully regulated to allow limited viscous relaxation prior to gelation. Moderately controlled reaction rates enable stress redistribution during network solidification and help maintain continuous interfacial contact despite volumetric contraction [84].

Taken together, these coupled chemical, mechanical, and kinetic processes indicate that the interface formed in in situ polymerized composite electrolytes should be interpreted as a reaction‐constructed interphase architecture. The final interfacial configuration is governed by monomer transport behavior, polymer chain propagation dynamics, and surface chemical interactions rather than by simple phase juxtaposition. Such reaction‐regulated interphase formation provides the fundamental physical basis for reaction‐mediated interfacial encapsulation and long‐term boundary stabilization in composite electrolyte systems, while simultaneously ensuring continuous ionic conduction across heterogeneous electrochemical interfaces.

Inorganic fillers embedded within composite electrolytes introduce additional complexity to interfacial evolution [85]. Surface chemistry, catalytic activity, and intrinsic ionic transport properties of filler particles can collectively influence local polymerization kinetics as well as interfacial chemical coupling behavior. Depending on their electrochemical and chemical functionalities, fillers may either act as passive structural modifiers of the polymer network or actively participate in ion transport reconstruction and interfacial stabilization. The mechanistic roles of active and non‐active fillers during in situ polymerization will be further discussed in the following section [86].

### 2.4 Active and Non‐Active Fillers: Mechanistic Roles During In Situ Polymerization

In addition to governing interfacial formation, inorganic fillers embedded in composite electrolytes may also influence the physicochemical processes occurring during in situ polymerization. Depending on their intrinsic ionic conductivity and electrochemical activity, these fillers are generally classified into two categories: active fillers, which possess intrinsic lithium‐ion conductivity and can directly participate in ion transport, and non‐active fillers, which are electrochemically inert and primarily modify the polymer matrix through structural or interfacial effects [87].

Active fillers typically refer to solid‐state lithium‐ion conductors such as oxide, sulfide, or halide electrolytes [88]. When incorporated into an in situ polymerizing system, these materials can simultaneously serve as inorganic ion‐transport media and interfacial anchoring sites for the polymer matrix. Their surfaces often contain coordinatively unsaturated metal centers or polarizable anionic frameworks that interact with polymer functional groups and lithium ions during polymerization. As a result, polymer chains may grow conformally along filler surfaces, generating chemically coupled soft–hard interfaces that facilitate lithium‐ion migration across the polymer–inorganic boundary. In such systems, the inorganic phase does not merely reinforce the polymer matrix but becomes an integral component of the ion‐transport network within the composite electrolyte [89, 90].

By contrast, non‐active fillers—including oxides such as SiO_2_, Al_2_O_3_, or TiO_2—_generally lack intrinsic lithium‐ion conductivity and therefore do not directly contribute to long‐range ion transport [91]. Their primary functions are associated with structural modification of the polymer network, including enhancement of mechanical strength, suppression of polymer crystallization, and regulation of polymer segmental dynamics. Surface functional groups on these particles may also interact with polymer chains through hydrogen bonding or Lewis acid–base interactions, thereby altering local polymer mobility and indirectly influencing ionic conductivity. Although these effects have been widely investigated in conventional composite polymer electrolytes, the fundamental mechanisms associated with non‐active fillers are relatively well established [92].

Given these distinctions, recent studies on in situ polymerized composite electrolytes have increasingly focused on systems incorporating active inorganic solid electrolytes, where polymerization occurs in the presence of lithium‐ion–conducting particles. In such systems, the polymer matrix and inorganic phase jointly construct a hybrid ion‐transport framework, and the resulting electrochemical performance is determined by the coupled evolution of polymer networks, filler interfaces, and lithium‐ion conduction pathways. Consequently, the present review primarily emphasizes composite electrolytes formed by polymer monomers and lithium‐ion–conducting inorganic electrolytes, while discussions of non‐active fillers are limited to their mechanistic differences in polymerization behavior and structural regulation.

The presence of active fillers further introduces an important consequence for lithium‐ion migration. Because both the polymer matrix and the inorganic phase can support ionic conduction, the resulting transport pathways become significantly more complex than those in conventional polymer electrolytes. The polymerization process, interfacial coupling, and spatial distribution of conductive particles collectively determine how lithium ions migrate through the composite electrolyte. Therefore, understanding how ion‐transport pathways are reconstructed in these in situ polymerized systems is essential for clarifying their electrochemical behavior. This topic will be discussed in detail in the following section.

### 2.5 Ion Transport Pathway Reconstruction in In Situ Systems

The incorporation of lithium‐ion–conducting fillers fundamentally alters the architecture of ion migration in in situ polymerized composite electrolytes. Unlike conventional polymer electrolytes, where lithium ions predominantly migrate through polymer segmental motion within a single continuous phase, in situ composite systems generate a heterogeneous transport environment composed of polymer domains, inorganic conductive particles, and dynamically formed interfacial regions [93]. As a result, lithium‐ion migration no longer follows a single dominant pathway but instead occurs through multiple coupled transport channels whose relative contributions are determined by the evolving microstructure of the composite electrolyte [94].

Within the polymer phase, lithium ions migrate primarily through coordination–decoordination processes involving polar functional groups along the polymer backbone. Ether oxygen, carbonyl, or nitrile groups can coordinate with Li^+^, allowing the ion to hop between neighboring coordination sites as polymer segments undergo thermally activated motion [95]. This mechanism, commonly referred to as polymer bulk transport, remains an essential conduction pathway in in situ polymerized electrolytes. However, polymerization‐induced crosslinking and network formation impose additional constraints on segmental mobility. As crosslink density increases during curing, the mobility of polymer chains becomes progressively restricted, which can elevate the activation energy for lithium‐ion hopping within the polymer matrix. Consequently, the polymer bulk phase alone often cannot sustain sufficiently high ionic conductivity once the network becomes mechanically rigid [96].

The presence of lithium‐ion–conducting fillers introduces a second transport channel, namely intra‐filler transport within the inorganic phase [97]. Active fillers possess well‐defined crystalline or amorphous frameworks that intrinsically support fast lithium‐ion migration. When these particles are dispersed within the polymerizing system, they act as embedded ionic conductors that provide alternative pathways for long‐range Li^+^ migration. If the spatial distribution of these particles approaches a percolation threshold, lithium ions may traverse the composite electrolyte by partially bypassing the polymer matrix and migrating through interconnected inorganic domains. In such cases, the composite electrolyte behaves as a hybrid ionic conductor in which both polymer and inorganic phases contribute to overall conductivity [98].

Between these two phases, a third and often decisive pathway emerges at the polymer–filler interface. Interfacial transport arises from the unique physicochemical environment formed where polymer chains, lithium salts, and inorganic surfaces converge [99]. Polymer segments anchored to filler surfaces frequently exhibit modified segmental dynamics compared with the bulk polymer phase. At the same time, surface lithium sites and polarizable lattice structures of inorganic fillers can locally modify Li^+^ coordination environments. These interfacial regions therefore possess distinct ion solvation structures and migration barriers relative to either pure polymer or bulk inorganic phases. In many composite electrolytes, lithium‐ion mobility along such interfacial regions can exceed that of the surrounding polymer matrix, effectively forming fast ion‐conduction corridors that bridge polymer and inorganic domains [100].

Importantly, these three transport pathways—polymer bulk transport, intra‐filler transport, and interfacial transport—do not emerge independently but are dynamically constructed during the polymerization process itself [101]. At the early stages of in situ polymerization, low‐viscosity precursor solutions enable homogeneous dispersion of fillers and intimate wetting of particle surfaces. Lithium salts and monomers distribute uniformly throughout the composite structure, establishing the initial ionic environment. As polymerization proceeds and network formation progresses, polymer chains gradually immobilize around filler particles and electrode surfaces, thereby defining the geometry of the interfacial regions. The spatial connectivity of inorganic particles and the degree of polymer confinement around them ultimately determine whether ion transport is dominated by polymer hopping, interfacial conduction, or filler‐mediated pathways [102].

Therefore, lithium‐ion conduction in in situ polymerized composite electrolytes should be understood as a reaction‐constructed transport network rather than a static structural property. The polymerization kinetics, filler distribution, and interfacial chemical interactions collectively determine the relative weight of each transport channel and thus govern the macroscopic ionic conductivity of the system. Designing high‐performance in situ composite electrolytes therefore requires simultaneous regulation of polymer segmental mobility, interfacial ion coordination environments, and inorganic phase connectivity. Only through such integrated structural control can continuous and low‐resistance lithium‐ion transport pathways be established across the heterogeneous architecture of in situ polymerized electrolyte systems.

Following the mechanistic discussion of polymerization kinetics, interfacial evolution, and ion transport pathways in Section 2, the subsequent sections focus on representative material systems in which these fundamental principles are manifested. In this review, in situ polymerized composite electrolytes are classified according to the molecular structure of the polymerizable monomers, rather than solely by polymerization mechanisms or filler types. This structure‐oriented framework is adopted because the molecular architecture of monomers fundamentally governs the polymerization behavior, segmental mobility, network formation, and interfacial interactions with inorganic fillers. As a result, different monomer families often exhibit distinct polymerization pathways, interfacial evolution characteristics, and ion transport behaviors in composite electrolytes. Based on this perspective, the following sections discuss two major categories of monomer systems: unsaturated monomers and cyclic monomers, highlighting how their structural features influence the construction and electrochemical performance of in situ polymerized composite electrolytes.

## 3 Unsaturated Monomers

Unsaturated monomers are integral to the synthesis of in situ polymerized electrolytes [103, 104]. These bonds, classified as “unsaturated” due to their incomplete saturation of carbon atoms, are highly reactive and readily participate in addition reactions with radicals or other molecules. This reactivity underpins the formation of extended polymer chains. Notably, double bonds provide abundant active sites for polymerization while introducing stereochemical specificity. Polymerization can proceed through free radical polymerization, where the double bond is attacked by a free radical to initiate chain growth, or ionic addition, where an ion reacts with the double bond to propagate the polymer chain. By strategically engineering monomer structures, polymers with tunable physicochemical properties can be constructed, including 3D networks generated through in situ polymerization to meet specific performance requirements [105, 106]. Representative polymer systems such as PMMA, PAN, PVDF and PEGMA will be discussed in detail in the following sections.

### 3.1 PMMA

**FIGURE 3:**
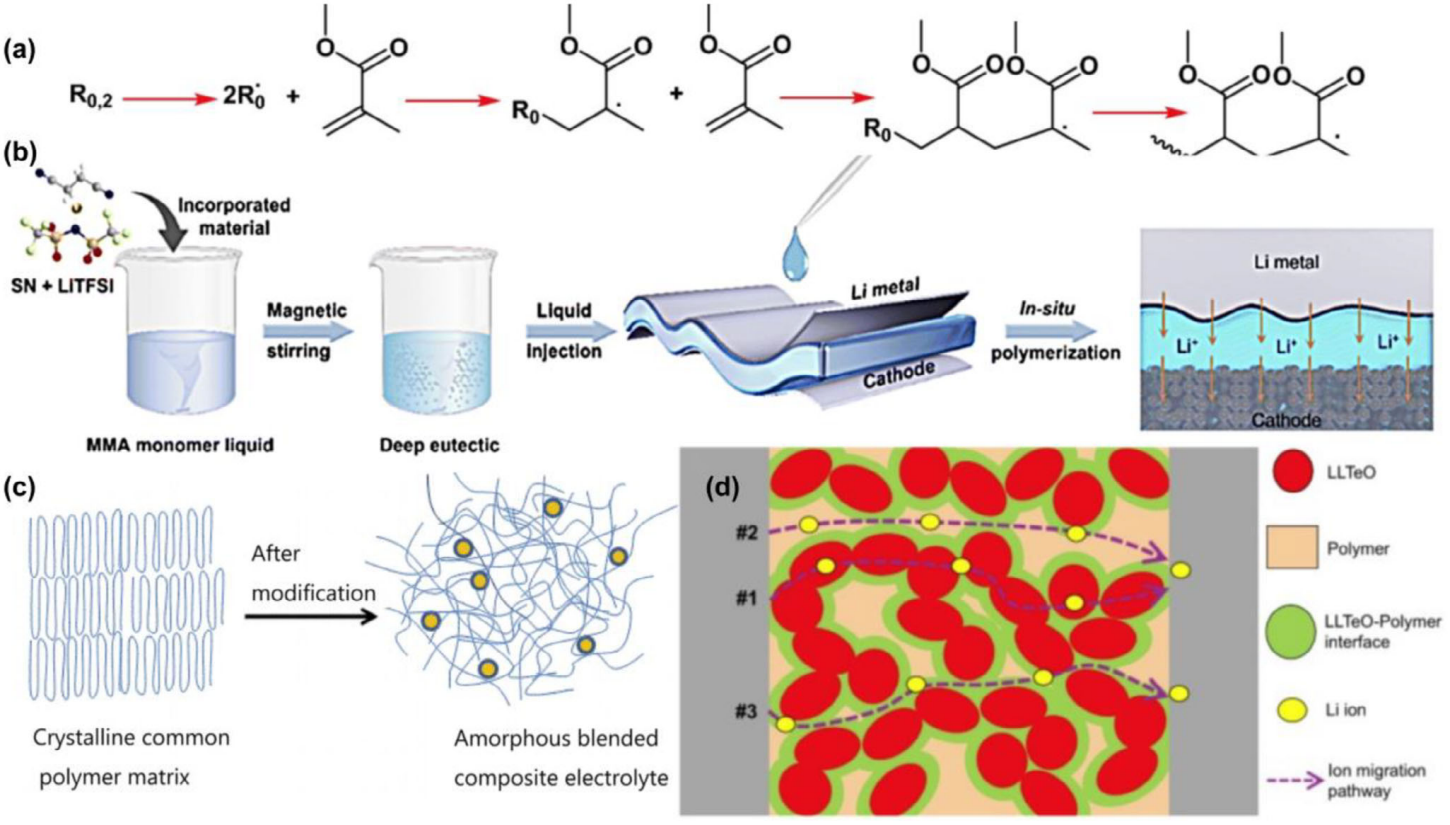
Representative polymerization mechanisms and structural engineering strategies for PMMA‐based electrolytes. (a) An illustration of the polymerization by free radical (FRP) of methyl methacrylate; Reproduced with permission from [109]. Copyright 2024, Springer Nature. (b) in situ fabricated PMMA‐based DEE elastomeric electrolyte exhibiting high ionic conductivity and stable SEI formation [119]. Reproduced under the terms of the Creative Commons CC BY 3.0 International License (https://creativecommons.org/licenses/by/3.0/). (c) Nano‐SiO2@PMMA‐doped composite polymer PVDF‐HFP/PMMA/PEO electrolyte for lithium metal batteries. Reproduced with permission from [120]. Copyright 2020, Springer Nature. (d) Dendrite‐suppressing PMMA/PVDF‐LLTeO‐70 PIC CPEs with high ionic conductivity. Reproduced with permission from [118]. Copyright 2024, Elsevier.

Poly (methyl methacrylate) (PMMA) is polymerized from methyl methacrylate (MMA) monomers via the reaction of carbon‐carbon double bonds (C═C) [107, 108]. PMMA is primarily synthesized through free‐radical polymerization, a process that involves four key stages: initiation, chain growth, chain transfer, and termination. In this method, free radicals initiate the polymerization by attacking the double bond of MMA monomers, leading to the growth of polymer chains, as illustrated in Figure 3a [109]. Guided by the spatial arrangement of MMA monomers, the chain‐growth polymerization process is highly directional, typically resulting in a linear chain structure [110]. This specific configuration imparts molecular stability to PMMA, while concurrently rendering it inherently electrically insulating due to the absence of mobile charge carriers. Previous studies have reported that pristine PMMA exhibits an exceptionally low electronic conductivity, generally ranging from 10^−16^ to 10^−15^ S cm^−1^ [111], which is a direct consequence of its insulating polymer backbone. These conductivity values underscore the lack of mobile charge carriers in PMMA, which is consistent with its low ionic conductivity of approximately 10^−6^ S cm^−1^ [107, 112]. To address the limitations of ionic conductivity, PMMA can be modified by incorporating lithium salts. This allows for the in situ synthesis of solid‐state electrolytes. Multiple carbonyl groups, either on the same PMMA chain or on different ones, can coordinate with a single Li^+^ ion, creating a chelate‐like coordination environment around the cation. This coordination not only stabilizes the Li^+^ within the polymer matrix but also enhances the dissociation of lithium salts by aiding in the separation of Li^+^ from the associated anions [113].

PMMA's compatibility with polar solvents, such as carbonate‐based solvents, facilitates the development of uniform gel and composite electrolytes. However, the physical crosslinking that occurs due to hydrogen bonding between polymer chains introduces structural rigidity, which reduces chain mobility and impacts film flexibility [114]. Additionally, the stable chain structure and relatively high glass transition temperature of ∼80^°^C further restrict segmental motion, thus limiting its ionic conductivity [115, 116]. To address these challenges, researchers have combined PMMA with various inorganic fillers and highly polar components to create composite electrolyte systems, with examples given below [117, 118].

#### 3.1.1 PMMA‐Based In Situ Polymerized Oxide Composite Electrolytes

The in situ polymerization process of the PMMA‐oxide composite solid‐state electrolyte is shown in the Figure 3b [119]. While PMMA does not serve as the primary Li^+^ conductor, it plays an indispensable role in establishing continuous ion‐transport pathways bridging the polymer network and the inorganic solid electrolyte particles. Specifically, the carbonyl groups (–C ═ O) along the PMMA chains engage in dipole–dipole interactions and weak hydrogen bonding with surface moieties, as displayed in Figure 3c, including lattice oxygen atoms on oxides such as LLZO [120]. This interfacial coupling significantly reduces the interfacial resistance from >10 000 to <500 Ω cm^2^ by facilitating Li transfer across the organic–inorganic interface. Furthermore, the ester oxygen atoms in PMMA weakly coordinate with Li^+^ (binding energy of ∼0.3 eV as revealed by DFT), which locally modifies the Li^+^ solvation structure and lowers the energy barrier for Li^+^ hopping into the oxide phase. These intermolecular and coordination effects promote the formation of a coherent interfacial buffer layer surrounding the oxide particles, maintaining good ionic contact between rigid oxide grains and providing continuous bridges for Li^+^ hopping across the oxide phase, as shown in Figure 3d [118]. The composite electrolyte achieves a high ionic conductivity of 10^−4^ S cm^−1^ at room temperature, showing an increase of several orders of magnitude over pure PMMA.

Moreover, the PMMA matrix could help to render a more uniform Li^+^ flux [121, 122]. Its polar chains help to adsorb and distribute anions, thereby suppressing space charge layer effects at the Li metal–electrolyte interface, resulting in stable cycling for over 1000 h in Li|Li symmetric cells at 0.2 mA cm^−2^ [121]. While the formation of a LiF and Li_2_O‐rich organic–inorganic composite SEI, promoted by the interfacial reaction between PMMA and the Li anode, enhances interfacial stability, leading to a Coulombic efficiency of >99.3% in Li|Cu half‐cells [123]. Structurally, the PMMA matrix creates a continuous organic phase that binds oxide particles, mitigating their inherent brittleness. This integration enhances the composite's flexibility, achieving a maximum stress of 155.2 MPa and strain of 182%, accommodating the volumetric strain of oxide particles during cycling, which helps prevent crack propagation [121].

#### 3.1.2 PMMA‐Based In Situ Polymerized Sulfide Composite Electrolytes

**FIGURE 4:**
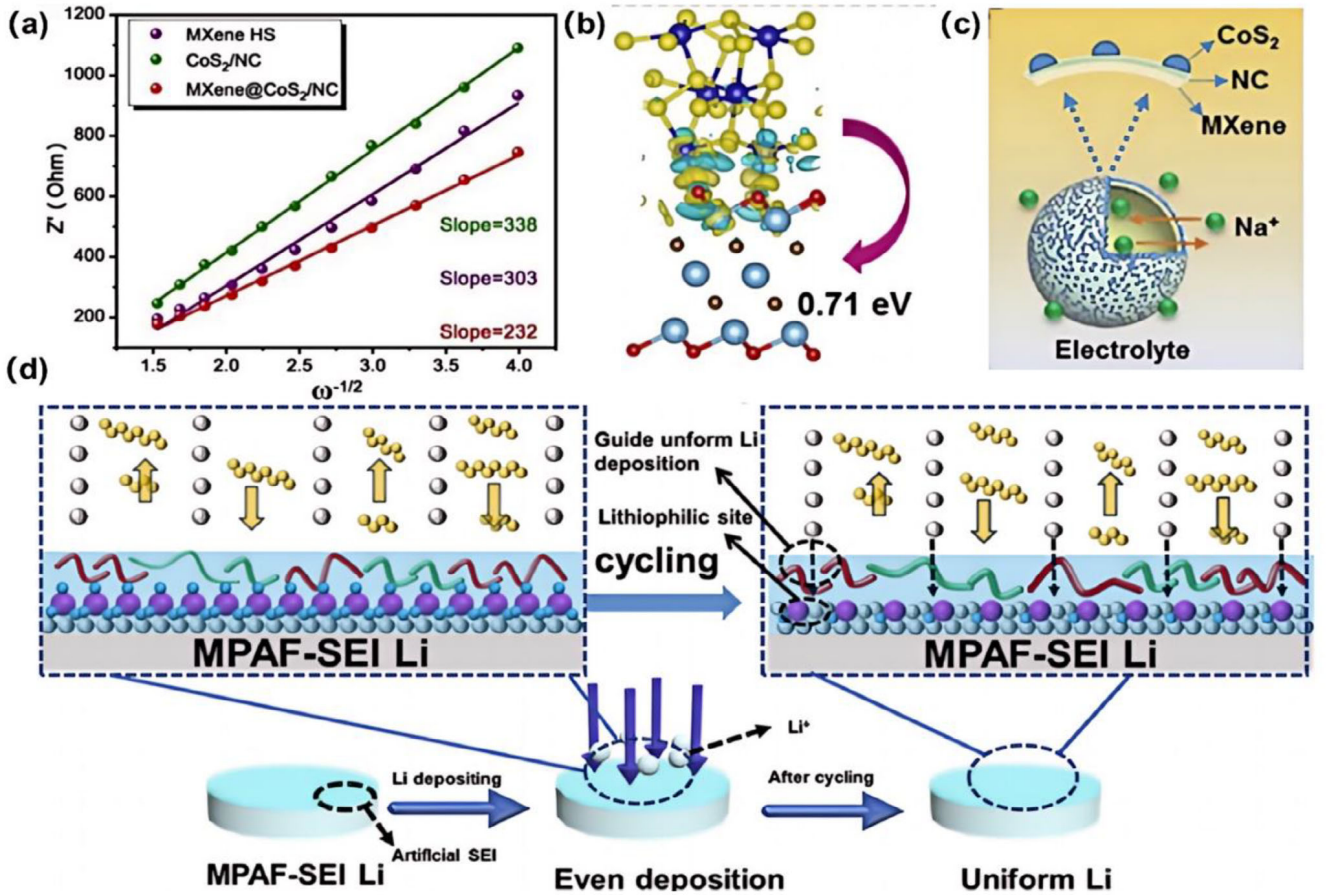
(a–c) Optimized Co─S bonds energy and confinement effect of hollow MXene@CoS_2_/NC for enhanced sodium storage kinetics and stability. Reproduced with permission from [128]. Copyright 2022, Elsevier. (d) In situ formed PMMA/PPC‐AlF_3_ artificial SEI (MPAF‐SEI) enabling a dendrite‐free and corrosion‐resistant Li‐S battery [129]. Reproduced under the terms of the Creative Commons CC BY 4.0 International License (https://creativecommons.org/licenses/by‐nc‐nd/4.0/).

While PMMA exhibits limited intrinsic ionic conductivity due to its covalent bonding network, it plays a crucial structural reinforcement role in sulfide‐based composite electrolytes. PMMA modulates the composite's 3D framework, indirectly enhancing Li^+^ transport by (1) optimizing morphology for continuous conduction pathways between the polymer matrix and sulfide electrolyte particles, (2) improving mechanical robustness via chain crosslinking, and (3) stabilizing electrode–electrolyte interfaces through chemical interactions, as shown in Figure 4 [124, 125, 126]. The carbonyl oxygen in PMMA's ester groups (‐COO‐) establishes dynamic coordination with Li^+^ through lone pair electron sharing, partially replacing sulfide‐based solvation environments. This weak interaction compresses Li^+^ solvation shells (2.5 to 1.8 Å), reduces desolvation energy barriers (0.45 to 0.28 eV), and increases Li^+^ transference numbers (t Li^+^: 0.3 to 0.65), as demonstrated in Figure 4a–c [127, 128]. Simultaneously, the polarity of the PMMA backbone electrostatically anchors bulky anions (e.g., PS_4_^3−^, TFSI^−^), suppressing concentration polarization by restricting anion mobility. This dual mechanism elevates free Li^+^ concentration from 5 × 10^20^ to 1.2 × 10^21^ cm^−3^ [129].

Incorporating sulfide particles (e.g., Li_7_P_3_S_11_) within high‐Tg PMMA matrices creates nanoscale confined regions that guide Li^+^ transport, lower interfacial reaction barriers, and improve synthesis efficiency. In these confined spaces, Li^+^ migrates via a hopping mechanism along sulfide grain boundaries, reducing activation energy (0.35 to 0.18 eV) and achieving a high conductivity of 2 × 10^−3^ S cm^−1^ (vs 1 × 10^−2^ S cm^−1^ for pure sulfides) [130]. Additionally, solvent‐induced phase separation generates hierarchical pore structures, where micropores (10 to 50 nm) localize Li^+^, and mesopores buffer mechanical stress, reducing deposition overpotentials from 50 to 15 mV [131]. Mechanically, the high modulus of sulfide electrolytes is complemented by the high viscoelasticity of PMMA (elongation at break >50%), endowing composites with both elasticity and toughness. This synergy disperses interfacial stress, suppresses dendrite penetration, improves cycling stability, and boosts the critical current density from 1.0 to 3.5 mA cm^−2^ [132]. At the interface, PMMA's ester groups enhance the elastic modulus of the SEI by forming an organic–inorganic composite layer rich in Li_2_CO_3_ and Li‐O‐C, achieving 5 GPa and ensuring compatibility with Li_3_P/Li_2_S phases, as displayed in Figure 4d. PMMA's stable chain structure resists volumetric fluctuations, inducing localized stress that distorts sulfide lattices, generating Li^+^ vacancies and increasing ionic conductivity by 15% under 1% strain [133]. Its hydrophobicity (contact angle >80°) further prevents sulfide hydrolysis, reinforcing interfacial stability [134].

Meanwhile, processing of PMMA/sulfide faces challenges. Hygroscopic solvents like THF and DMF introduce residual moisture, undermining the stringent dry conditions (<1 ppm H_2_O) required for sulfides. Additionally, high processing temperatures (>80°C) and pressures risk nucleophilic substitution between PMMA ester groups and sulfide S^2−^, forming interfacial‐degrading thioesters. PMMA's oxidation potential (∼4.2 V vs. Li^+^/Li) also nears that of high‐voltage cathodes (e.g., NMC811, 4.3 V), making it easy to decompose at high voltages [135, 136]. Moreover, sulfide particle aggregation via van der Waals interactions disrupts continuous ion‐conducting pathways, reducing composite conductivity to one‐tenth of that of pristine sulfides.

#### 3.1.3 PMMA‐Based In Situ Polymerized Halide Composite Electrolytes

The methyl methacrylate units in PMMA contain highly polar carbonyl (C ═ O) groups. The lone pairs on the carbonyl oxygen can act as Lewis bases and interact with the electropositive regions of halogen atoms through weak electrostatic or Lewis acid–base interactions originating from electronegativity differences. Such interactions can modulate interfacial affinity between the polymer matrix and halide‐based fillers, thereby influencing local ion transport pathways [137]. Additionally, the side chains of PMMA offer modification sites for halogen incorporation through covalent bonding or non‐covalent interactions. Moreover, the electron‐withdrawing effect of halogens induces an uneven electron distribution within the polymer, generating a stronger molecular dipole moment which enhances both the overall polarity and the dielectric constant of the polymer [138]. A higher dielectric constant helps screen electrostatic interactions between ions, facilitating lithium salt dissolution and ion dissociation [139]. As a result, halogenated solid‐state electrolytes based on PMMA composites can (1) facilitate ion transport, (2) mitigate hydrolysis‐induced side reactions, and (3) enhance long‐term cycling stability [129, 140].

Specifically, Li^+^ can coordinate with both the carbonyl oxygen of PMMA and Cl^−^ in the halide lattice, forming a Li‐O‐Cl ternary structure with relatively weak coordination. This reduces the Li^+^ desolation energy barrier (from 0.45 to 0.25 eV) [141], thereby improving ion transport at the interface. Additionally, under an applied electric field, the ester dipoles of PMMA align along the halide surface, generating a localized electric field (0.5 V nm^−1^) that accelerates Li^+^ migration at the interface. Furthermore, the hydrophobicity of PMMA effectively encapsulates halide particles, preventing their exposure to moisture and significantly mitigating hydrolysis‐induced side reactions (reducing HCl release from >100 to <10 ppm). Finally, the smooth and uniform surface of the PMMA composite electrolyte membrane enhances the cycling stability and consistency of the battery, minimizing ion transport hindrances caused by surface roughness [142].

Despite the advantages, PMMA‐halide composite solid electrolytes face significant challenges. First, the combination of PMMA and halide with low conductivity limits the overall conductivity of the composites. The lack of strong coordination or ion‐exchange interactions between PMMA and halide ions results in only a marginal increase in ionic conductivity [143]. Second, while PMMA encapsulation smooths the halide electrolyte surface, its relatively low dielectric constant (ε_r_ ∼ 3.5) leads to a large mismatch with halides (e.g., Li_3_YCl_6_, ε_r_ ∼ 25) [144]. This mismatch induces a spatial charge layer up to ∼10 nm thick, where Li^+^ accumulates on the halide side and anions (e.g., Cl^−^) concentrate on the PMMA side. Furthermore, halides, with their rigid crystal lattice, rely on Li^+^ vacancy or interstitial conduction mechanisms. Meanwhile, PMMA's high glass transition temperature restricts segmental motion, preventing the formation of a synergistic “lattice hopping–segmental migration” transport pathway. Thus, the activation energy for ion conduction (E_a_) can reach as high as 0.5 eV. Finally, when paired with high‐voltage cathodes, PMMA is prone to oxidation, generating carboxylic acid derivatives that accelerate interfacial resistance buildup [145, 146, 147].

From a processing perspective, the high elastic modulus of halides (e.g., Li_3_InCl_6_ ∼25 GPa) results in the formation of rigid interfaces with PMMA during film fabrication. This rigidity leads to localized stress accumulation during charge–discharge cycling, causing crack propagation and eventual mechanical failure of the electrolyte. Additionally, the poor solubility of halides in PMMA makes it challenging to fabricate composite electrolytes using conventional solution or melt‐processing methods. Even if a uniform film is achieved, PMMA's inability to undergo viscoelastic deformation prevents it from effectively absorbing stress from Li deposition and stripping, leading to a low critical current density. Furthermore, residual moisture from processing solvents may trigger halide hydrolysis (e.g., Li+YCl_6_ + H_2_O → LiOH + Y(OH)_3_ + HCl), analogous to the instability observed in sulfide‐based composites [148]. Therefore, further research efforts should focus on addressing these challenges.

### 3.2 PAN

Polyacrylonitrile (PAN) is a widely used polymer whose backbone comprises of repeating acrylonitrile units (–CH_2–_CH(CN)–) [149, 150]. The strongly polar nitrile groups (–CN) can coordinate with Li^+^, forming labile complexes that facilitate Li^+^ solvation and enhance ionic mobility, thereby endowing PAN with latent lithium conducting capability. Moreover, the–CH_2–_CH(CN)– motif shows great chain rigidity and mechanical strength. Through appropriate crosslinking, PAN can be fabricated into robust solid electrolyte membranes with improved cycle stability. Additionally, the intrinsic thermal resilience of PAN enables these membranes to maintain structural integrity at elevated temperatures.

On the other hand, in the absence of plasticizers, the intrinsic ionic conductivity and Li^+^ transference for pure PAN remain limited due to the high chain rigidity that constrains segmental motion and reduces the degrees of freedom necessary for efficient ion transport. A further impediment for the lithium ion transport arises from the relatively high crystallinity of PAN which markedly hinder Li^+^ diffusion. To overcome these limitations, plasticizers such as polyethylene oxide (PEO) or polyethyleneimine (PEI) are commonly incorporated [151]. These additives increase chain flexibility, reduce crystallinity, and generate more continuous ion conductive pathways. Various PAN‐based composite electrolyte architectures have been developed, as listed below [152].

#### 3.2.1 PAN‐Based In Situ Polymerized Oxide Composite Electrolytes

**FIGURE 5:**
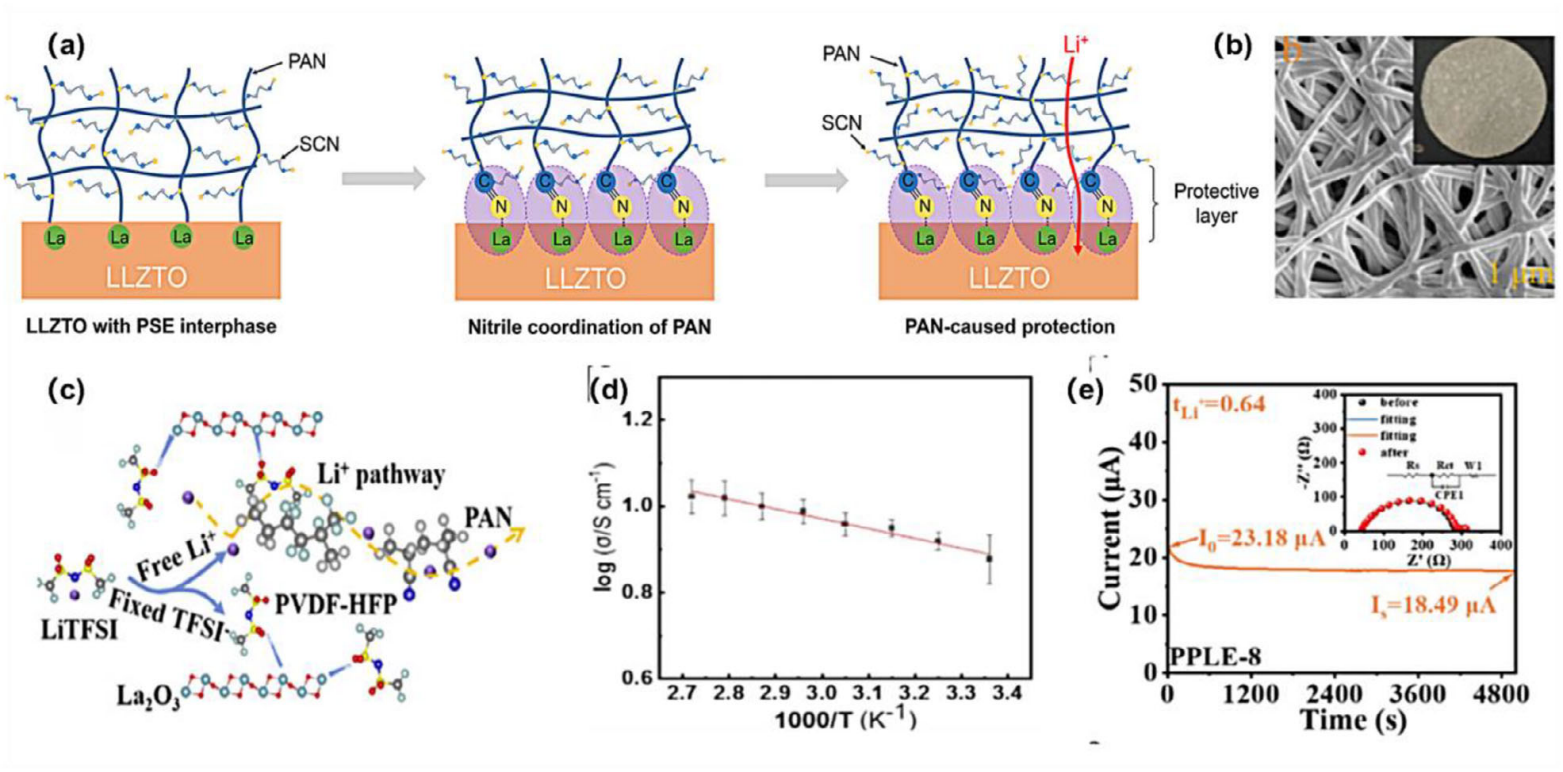
Enhanced interfacial conductivity and stability of PAN‐oxide composite electrolytes for solid‐state lithium batteries. (a) The function mechanism of PSE interphase on the surface of LLZTO electrolyte. Reproduced with permission from [154]. Copyright 2021, Wiley. (b) An electrospinning method to synthesize a 3D cross‐linkied PAN nanofiber framework and multiphase Al_2_O_3_ ceramic fillers. Reproduced with permission from [155]. Copyright 2025, American Chemical Society. (c) La_2_O_3_‐Reinforced Polymer Electrolyte with Enhanced Interfacial Lithium‐Ion Conductivity. Reproduced with permission from [161]. Copyright 2025, Wiley. (d) Ring‐opening polymerization reconfigures polyacrylonitrile network for ultra stable solid‐state lithium metal batteries. Reproduced with permission from [157]. Copyright 2024, Wiley. (e) An economic integrated self‐standing anode@quasi‐solid‐state electrolyte membrane. Reproduced with permission from [160]. Copyright 2025, Elsevier.

The introduction of plasticizers such as ethylene carbonate (EC) and propylene carbonate (PC) significantly enhances the compatibility between PAN and oxide‐based electrolytes, further improving the overall ionic conductivity of the composite electrolyte. More importantly, the highly polar –CN groups in acrylonitrile coordinate with Li^+^ in the oxide‐based electrolyte, increasing lithium‐ion solvation and reducing its strong attraction to the oxide surface, as displayed in Figure 5a [153, 154]. The molecular structure of PAN can effectively fill voids within the oxide matrix, as revealed in Figure 5b–d [155, 156, 157], facilitating interaction with the oxide surface and forming a more continuous ion transport pathway [158]. Additionally, modified PAN provides macroscopic flexibility, addressing the issue of ion migration limitations in rigid oxide electrolytes, particularly at low temperatures. Building on these theoretical foundations, Liu et al. developed a porous PAN‐LLZTO electrolyte membrane via in situ polymerization, showing a high ionic conductivity at room temperature with enhanced interfacial stability, attributed to the formation of dual stable interfaces (1.9 × 10^−3^ S cm^−1^) [159].

This structural flexibility provides oxide materials with more degrees of freedom during the composite process, enabling smoother lithium‐ion transport, the PAN‐oxide interaction homogenizes the electrolytes distribution and strengths the CSE. The synergistic effect of polar functional groups, polymer chains, and mechanical properties prevents Li from being immobilized within the oxide, reducing interfacial resistance and ultimately improving ionic conductivity, as presented in Figure 5e [157, 160].

#### 3.2.2 PAN‐Based In Situ Polymerized Sulfide Composite Electrolytes

**FIGURE 6:**
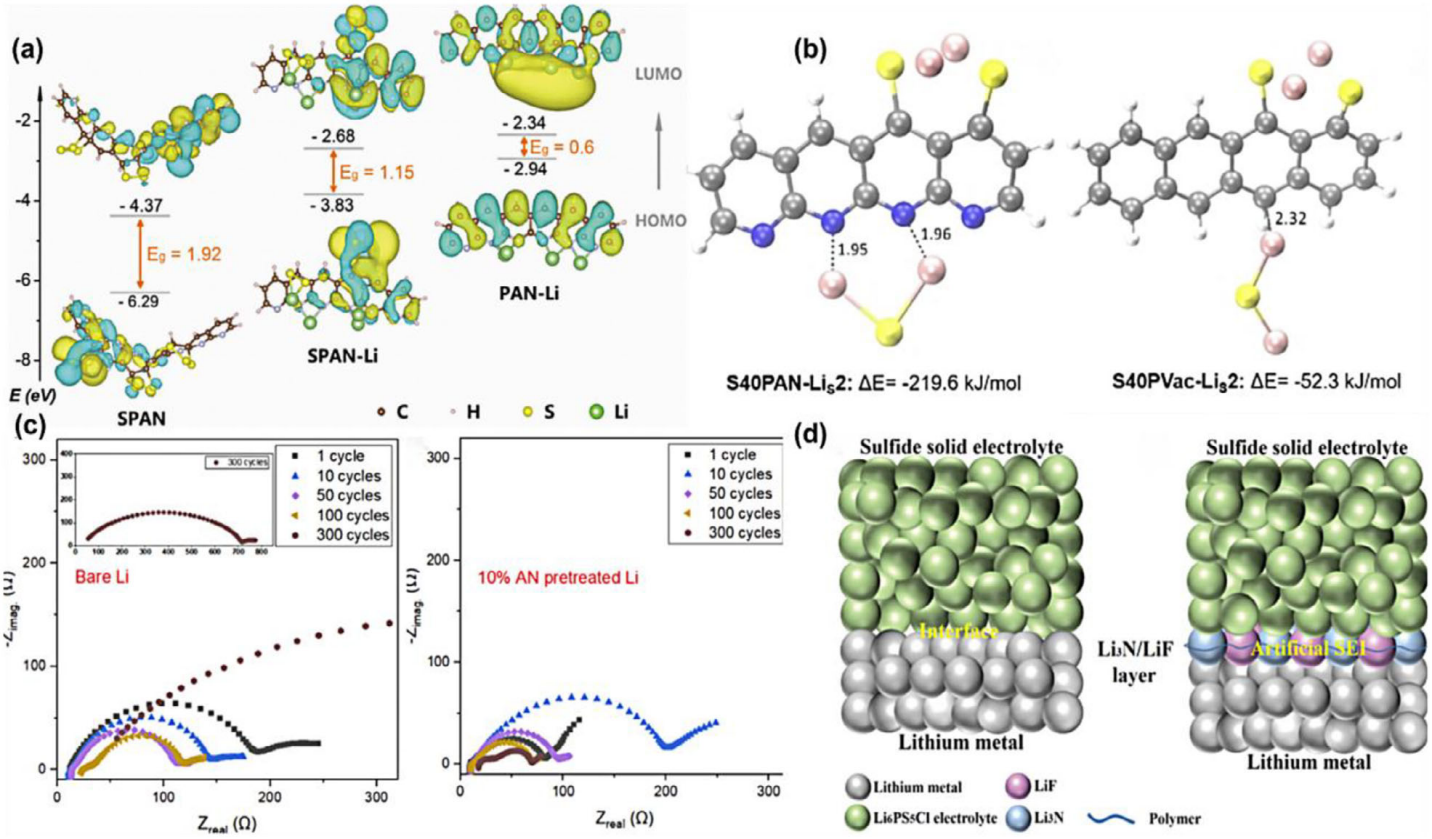
Structural and electrochemical properties of PAN‐based composite electrolytes with sulfide fillers for advanced lithium batteries. (a) The calculated HOMO/LUMO energy level diagrams of SPAN, partially lithiated SPAN (SPAN‐Li), and fully lithiated PAN (PAN‐Li) by frontier molecular orbital analysis. Reproduced with permission from [162]. Copyright 2019, Wiley. (b) A manganese cobalt sulfide‐doped fibrous sulfurized polyacrylonitrile for high‐rate and long‐life Li‐S batteries. Reproduced with permission from [163]. Copyright 2025, Royal Society of Chemistry. (c) A highly effective polyacrylonitrile‐rich artificial SEI. Reproduced with permission from [166]. Copyright 2024, American Chemical Society. (d) An in situ ion‐conducting protective layer strategy to stable lithium metal anode. Reproduced with permission from [167]. Copyright 2020, Wiley.

A similar enhancement effect is observed in PAN‐sulfide composite electrolytes. The highly polar –CN groups, owing to their strong electron‐withdrawing capability and large dipole moment, can establish stable ion–dipole interactions with Li^+^ in sulfide‐based electrolytes, as shown in Figure 6a,b [162, 163, 164]. When the interaction energy is moderate, it enables Li^+^ to “hop” between different coordination sites without becoming trapped, thereby reducing the activation energy for ion migration and promoting rapid charge transport. This synergy promotes the uniform distribution of Li^+^ within the electrolyte, optimizing ion transport pathways and forming a ‘bridge’ structure at the composite electrolyte–electrode interface [165]. As depicted in Figure 6c,d [166, 167], such an intermediate or transition layer at the interface helps promote Li^+^ migration, mitigating interfacial impedance caused by local aggregation or uneven ion distribution. Meanwhile, when PAN and pure sulfide electrolytes contact with a lithium metal anode, side reactions may occur, leading to an unstable interphase layer that increases resistance to Li^+^ transport. Therefore, careful control of the polymerization process is crucial to ensure interfacial stability.

#### 3.2.3 PAN‐Based In Situ Polymerized Halide Composite Electrolytes

While halides are inherently brittle, the incorporation of PAN can significantly enhance the mechanical properties of in situ polymerized electrolytes, improving their processability [168]. PAN forms a flexible matrix that encapsulates the halide particles, enhancing the contact between particles and preventing the interface from cracking or detaching due to stress during battery cycling. However, the strong ionic bonding in halides prevents the polar groups in PAN from forming strong coordination interactions, resulting in weak or unstable interactions between the two components. This issue is more evident for larger halide anions with lower charge density, such as I^−^, where the interaction with cyano groups is minimal and does not significantly promote lithium‐ion transport, leading to only limited improvements in ionic conductivity. Additionally, halides typically exhibit densely packed crystal structures at room temperature, with few ion migration pathways, while adding highly crystalline PAN can't improve the ion transport [169].

### 3.3 Fluorine‐Containing Monomers

Fluorine‐containing monomers have garnered significant attention in the development of advanced polymer electrolytes for lithium‐ion batteries. The incorporation of fluorine atoms into polymer backbones imparts unique properties such as high thermal stability, chemical resistance, and a wide electrochemical stability window [170]. From a structural perspective, fluorinated polymer systems provide a complementary design strategy to conventional unsaturated monomers by introducing highly polar C‐F bonds (∼485 kJ mol^−1^) that simultaneously regulate salt dissociation, interfacial stability, and electrochemical robustness in in situ composite electrolytes [171]. Common fluorinated monomers include vinylidene fluoride (VDF), trifluoroethylene (TrFE), and hexafluoropropylene (HFP), each contributing distinct structural and functional characteristics to the polymers [172]. Structurally, the presence of fluorine atoms increases the polarity and decreases the surface energy of the polymers, leading to improved dielectric properties and reduced solubility of lithium salts [173, 174]. These features facilitate better ion transport and compatibility with lithium metal anodes [174]. However, the high crystallinity often associated with fluorinated polymers can hinder ionic conductivity by limiting the amorphous regions necessary for efficient ion mobility. To address this, strategies such as copolymerization with non‐fluorinated monomers or the introduction of plasticizers are employed to disrupt the crystalline domains and enhance the amorphous content [175].

Among fluorinated polymers, poly (vinylidene fluoride) (PVDF) stands out due to its favorable balance of mechanical strength, thermal stability, and electrochemical performance. The subsequent section will discuss into the PVDF‐based electrolytes, exploring strategies to mitigate their limitations and enhance their suitability for next‐generation lithium‐ion batteries. Notably, although many PVDF‐based electrolytes have traditionally been prepared via solution casting, recent studies increasingly integrate fluorinated polymer frameworks into in situ constructed composite electrolytes, where polymer networks or interphases are generated directly within the electrolyte environment. In this context, PVDF and its derivatives serve not only as structural matrices but also as chemically functional components that regulate interfacial stability and ion transport. Therefore, fluorinated polymer frameworks such as PVDF remain representative systems for discussing the role of fluorine‐containing polymers in in situ composite electrolytes [176].

Poly (vinylidene fluoride) (PVDF), with the repeating unit (C_2_H_2_F_2_)_n_, is synthesized by the polymerization of the fluorinated monomer vinylidene fluoride [177]. As discussed previously, fluorine has a high electronegativity (∼4.0), which imparts strong polarity to the C─F bonds [178]. In PVDF, the C─F and C─H bonds are asymmetrically distributed along the polymer backbone, resulting in a net dipole moment of approximately 1.4 D and a high dielectric constant ranging from 8 to 12. The electron cloud in the C─F bond is significantly skewed toward the fluorine atom, leading to charge separation along the chain. This polarization plays a critical role in PVDF's chemical resistance and dielectric behavior.

Beyond its intramolecular dipolar character, PVDF also exhibits weak intermolecular hydrogen bonding between the partially positive hydrogen atoms and partially negative fluorine atoms on adjacent chains. These interactions influence the polymer's crystallization behavior by promoting interchain association and aggregation. Additionally, PVDF chains are stabilized by significant van der Waals forces, including dispersion, induction, and orientation forces, due to the dense arrangement of polar C─F and C─H bonds. These non‐covalent interactions collectively affect the thermal properties, solubility, and morphological stability of PVDF, governing its melting point, crystallinity, and molecular packing.

From a structural perspective, PVDF exhibits several crystalline polymorphs,α, β, and γ, determined by the spatial configuration of fluorine and hydrogen atoms along its polymer backbone [179]. The α phase is the most commonly observed and kinetically favored crystalline form of PVDF under conventional processing conditions such as melt crystallization or solvent casting. While it is frequently referred to as thermodynamically stable, this stability is context‐dependent and may vary with temperature and external stimuli. Characterized by a TGTG′ (trans–gauche–trans–gauche) helical conformation, its molecular chains arrange into lamellar layers via van der Waals interactions [180]. The alternating fluorine and hydrogen atoms lead to partial dipole cancellation, resulting in a non‐polar structure with moderate mechanical flexibility. This phase typically forms under standard processing conditions such as melt cooling or solvent casting.

In the β phase, PVDF chains exhibit a fully trans (TTTT) conformation, resulting in a compact, highly crystalline structure with a pronounced net dipole [181]. This phase shows a high dielectric constant, which facilitates lithium salt dissociation by mitigating ion‐pair interactions and promotes Li^+^ conduction (conductivity up to 10^−4^ S cm^−1^) [182]. The internal electric fields generated by the dipole alignment further enhance ion mobility. Nevertheless, the increased crystallinity also restricts segmental chain dynamics, limiting overall ion transport. The β phase is typically formed through uniaxial stretching, electric field orientation, or nanomaterial templating [183].

Conversely, the γ phase adopts a mixed T_3_GT_3_G’ conformation, integrating both trans and gauche units. This structural complexity imparts intermediate polarity and improved chain flexibility, contributing to reduced crystallinity and enhanced segmental dynamics. As a result, the γ phase often shows superior ionic conductivity at ambient temperatures compared to the α phase, while maintaining structural compliance. It can be obtained via solvent‐controlled crystallization or polymer blending, and is increasingly explored in the design of flexible, high‐performance solid polymer electrolytes.

Due to the inherent stability of C─F bonds and the specific molecular architecture of PVDF, it features a broad electrochemical stability window (above 4 V) It remains inert under high voltage, making it compatible with high‐voltage electrodes and suitable for high‐energy battery systems. The material also offers good thermal stability, with decomposition temperatures above 300°C, ensuring reliable performance across a wide temperature range. Furthermore, PVDF is insoluble in common carbonate electrolytes, making it ideal for constructing quasi‐solid‐state or all‐solid‐state systems [184].

Despite its advantages, PVDF has notable drawbacks. Its crystallinity is relatively high (35%–70%), and crystalline domains exhibit high ion transport activation energy (E_a_ > 0.5 eV), limiting conductivity [185]. Ion conduction occurs via a hopping mechanism that depends on chain mobility, which is significantly reduced at low temperatures due to its Tg of −35 °C. As a result, conductivity drops by 1–2 orders of magnitude at –20°C. Under high temperatures or voltages, C─F bond cleavage may occur, producing trace HF that corrodes electrodes and forms passivation layers like LiF, increasing interfacial resistance.

#### 3.3.1 PVDF‐Based In Situ Polymerized Oxide Composite Electrolytes

Introducing oxide fillers (e.g., Li_3_PO_4_, LLZO, Al_2_O_3_) into PVDF‐based solid electrolytes is an effective approach to improve ion transport, mechanical strength, and interface stability [186]. The electronegative fluorine atoms in PVDF electrostatically attract positively charged ions (such as Li^+^) or polar groups on oxide surfaces, enhancing adhesion between polymer and filler. This promotes uniform dispersion of oxide particles, reduces agglomeration, and minimizes interface defects, aiding lithium‐ion transfer at interfaces [187].

**FIGURE 7:**
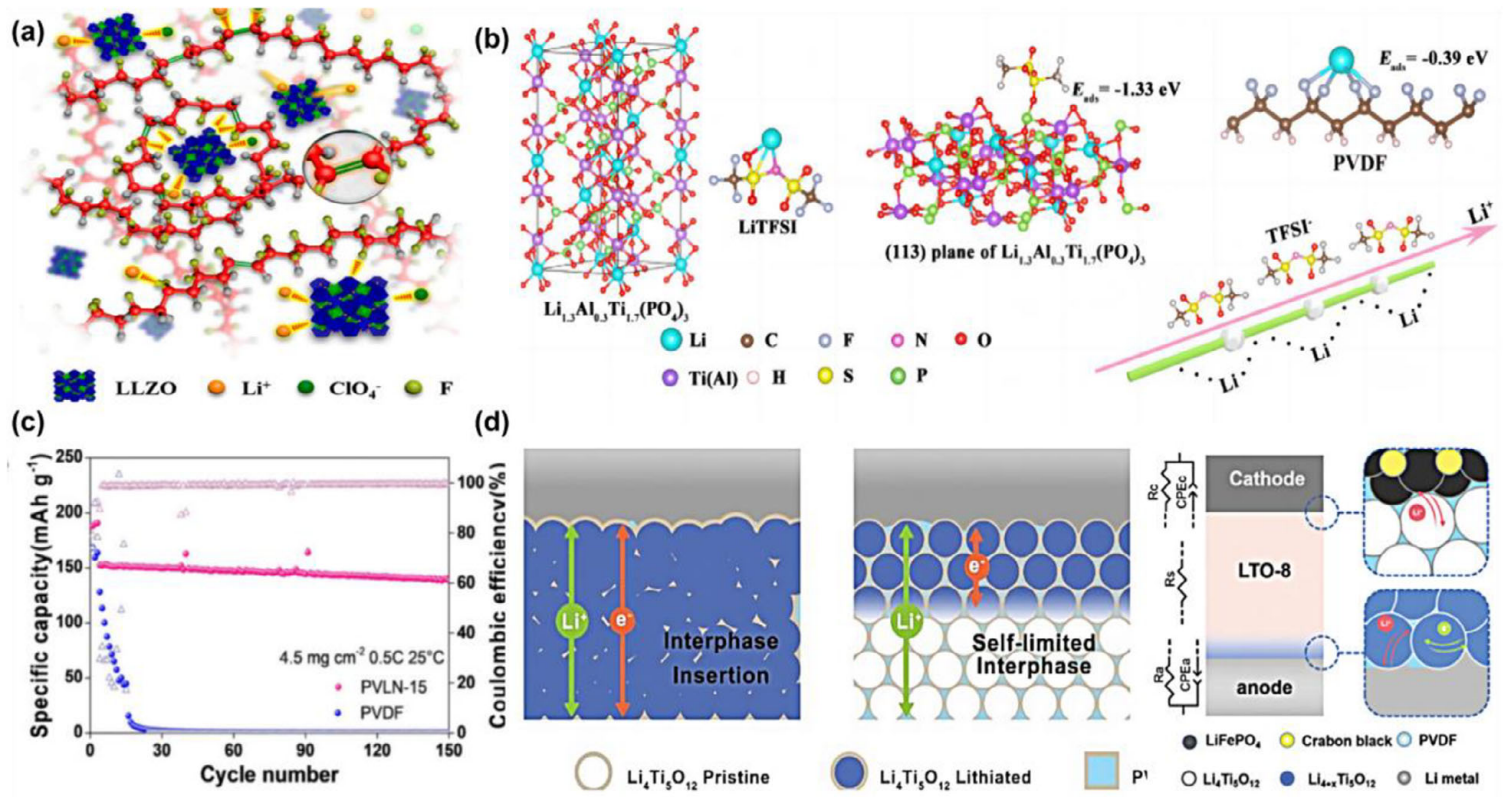
Synergistic coupling of PVDF with LLZTO and other oxide materials for enhanced ionic conductivity and interfacial stability in composite solid electrolytes. (a) Synergistic Coupling between LLZTO and PVDF. Reproduced with permission from [192]. Copyright 2017, American Chemical Society. (b) Constructing 3D Li‐percolated transport network in composite polymer electrolytes. Reproduced with permission from [191]. Copyright 2022, Elsevier. (c) A chemically stable ceramic‐polymer‐anchored solvent composite electrolyte. Reproduced with permission from [193]. Copyright 2021, Wiley. (d) A solid electrolyte based on Li_4_Ti_5_O_12_ with PVDF. Reproduced with permission from [194]. Copyright 2022, Elsevier.

PVDF contains fluorine atoms that interact electrostatically with oxide particles, such as Li_3_PO_4_ and LLZO, both of which exhibit inherent polarity. These interactions facilitate the formation of ion conduction channels within the composite material, as illustrated in Figure 7a. Additionally, weak hydrogen bonds between the fluorine‐hydrogen groups in PVDF and oxygen atoms in the oxides further enhances the material's ion conduction properties. This combination of electrostatic attraction and hydrogen bonding reduces interfacial resistance and enhances the uniform dispersion of oxide particles, directly boosting ion conductivity. As depicted in Figure 7b [188], the integration of a flexible PVDF matrix and conductive oxide particles generates a synergistic effect that significantly enhances ion conduction. PVDF provides amorphous regions for ion transport, while oxide particles, such as Li_3_PO_4_ and LLZO [189], offer high‐conductivity lithium‐ion channels. This collaborative interaction forms a polymer‐ceramic interface network that reduces activation energy and improves ion transport efficiency, as shown in Figure 7c [190, 191]. Furthermore, optimizing oxide particles can increases the effective interfacial contact area and forms continuous percolated networks, facilitating ion transport along the interface.

Optimizing the interface between PVDF and oxide particles is crucial for reducing interfacial resistance and enhancing ion migration, we can comprehend this more intuitively from Figure 7d [193, 194]. By establishing stronger polar interactions between PVDF and oxide surfaces, the overall stability of the composite is enhanced. A gradient structure, with oxide‐enriched layers near the electrode and PVDF enriched layers in the bulk, further optimizes both mechanical strength and ion transport across the interface. Furthermore, surface coatings on oxides, such as LiF, can prevent the formation of harmful decomposition products, thereby protecting the lithium metal anode and extending the lifetime of the electrolyte. In addition to improving interfacial adhesion and ion transport, oxide fillers also play an important role in regulating PVDF defluorination reactions. Under high voltage or elevated temperature conditions, partial C─F bond cleavage in PVDF may generate trace HF or fluoride species, which can degrade electrode–electrolyte interfaces. Recent studies have shown that oxide surfaces can effectively suppress or redirect such reactions through surface chemical interactions. Electron‐withdrawing metal centers on oxides can stabilize the polarized C─F bonds of PVDF, reducing the probability of bond cleavage. Meanwhile, oxide surfaces can act as scavengers for proton or fluoride intermediates generated during early stages of defluorination, thereby preventing the accumulation of corrosive HF species. Surface engineering of oxide fillers further enhances this regulation effect [195]. For example, LiF‐coated or Al_2_O_3_‐modified oxide particles can capture reactive fluorinated intermediates and convert them into stable interfacial species, effectively transforming a detrimental degradation pathway into a protective passivation process. The formation of LiF‐rich interphases at polymer–oxide boundaries has been reported to improve interfacial stability with lithium metal and suppress continuous electrolyte decomposition. Such strategies demonstrate that rational filler design can not only suppress PVDF defluorination but also harness controlled defluorination to construct stable ion‐conducting interfacial layers.

The flexible chains of PVDF enable the composite material to adapt to stress during charge‐discharge cycles, preventing the formation of cracks or detachment of oxide particles. This flexibility is crucial for maintaining the mechanical integrity of the composite over extended periods. The rigidity of oxides, such as LLZO, contributes to structural stability, reducing the likelihood of membrane rupture during cycling. The enhanced mechanical strength of the composite ensures long‐term stability, thereby contributing to sustained ion transport efficiency and reliability. Remarkably, Zhang's group combined experimental investigations with first‐principle calculations and discovered that La atoms on LLZO surfaces interact with nitrogen atoms in PVDF through Lewis acid‐base reactions, triggering PVDF defluorination and markedly enhancing the properties of flexible electrolyte membranes [192]. As a result, the CPEs achieved an impressive ionic conductivity of 5 × 10^−4^ S cm^−1^ at room temperature, and the assembled LiCoO_2_|CPEs|Li cells exhibited excellent cycling and rate stability.

#### 3.3.2 PVDF‐Based In Situ Polymerized Sulfide Composite Electrolytes

**FIGURE 8:**
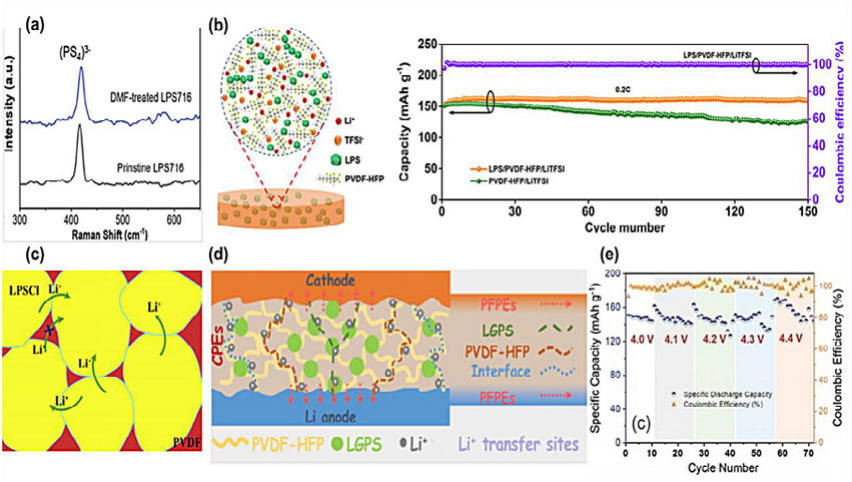
Structural and electrochemical performance of PVDF‐based composite solid electrolytes with embedded sulfide fillers for enhanced stability and dendrite suppression. (a) Free‐standing 78Li_2_S‐22P_2_S_5_ glass‐ceramic composite solid electrolyte membranes. Reproduced with permission from [196]. Copyright 2020, Elsevier. (b) A combination of inorganic sulfide Li_7_PS_6_ with PVDF‐HFP. Reproduced with permission from [197]. Copyright 2020, American Chemical Society. (c) LPSCl/PVDF composite electrolytes prepared by a slurry method. Reproduced with permission from [199]. Copyright 2020, Elsevier. (d) PVDF/LGPS composite solid‐state Electrolytes for dendrite‐free Li metal batteries. Reproduced with permission from [200]. Copyright 2020, Elsevier. (e) Embedding sulfides into PVDF/HFP polymer to enhance interfaical stability. Reproduced with permission from [201]. Copyright 2024, Wiley.

The strong polarity of the C─F bonds in PVDF allows interaction with sulfur ions (S^2−^) or phosphorothioate groups (PS_4_^3−^) on the surface of sulfides (e.g., Li_3_PS_4_, Li_7_P_3_S_11_) through dipole‐dipole interactions or weak hydrogen bonding, as evidenced in Figure 8a [184, 196]. Achieving a percolation threshold (>30% volume fraction) for sulfide nanoparticles such as Li_6_PS_5_Cl in the PVDF matrix is essential to forming continuous lithium‐ion transport pathways that span both the inorganic sulfide domains and the polymer matrix. Improved nanoparticle dispersion achieved through high‐energy ball milling or solution blending facilitates the formation of continuous hybrid conduction channels at polymer‐sulfide interfaces, enabling the composite electrolyte to reach ionic conductivities on the order of 10^−4^ S cm^−1^. The amorphous regions of PVDF, with a glass transition temperature, allow enhanced chain segmental movement upon heating. This results in a ‘relay‐style’ transmission mechanism, where Li^+^ migrates between sulfide particles and over long distances through polymer chain movement, as demonstrated in Figure 8b–d [197, 198, 199]. This interaction reduces E_a_ from 0.5 eV to below 0.3 eV, enhancing ion conductivity.

The high electronegativity of fluorine further enhances electrostatic attraction, improving interface wettability and reducing interfacial voids, thus stabilizing the interface and improving conductivity. Surface coatings, such as fluorosilane coupling agents or LiF transition layers, enhance compatibility between PVDF and sulfides [202], reducing interfacial resistance from >1000 to <500 Ω·cm^2^. [184] Wang et al. reported a typical example by embedding LPSC into a PVDF‐HFP matrix, achieving excellent performance mentioned above (like chemical stability and ionic conductivity).This study provides valuable insight into balancing mechanical and electrochemical properties in flexible composite electrolytes [197].

The C─F bonds in PVDF contribute to synergistic passivation by forming a LiF containing SEI layer on the lithium metal surface, while the PS_4_^3−^ groups in sulfides produce passivating products such as Li_3_P and Li_2_S. These components create a dense interface layer that suppresses side reactions, reducing interfacial impedance from >2000 to <800 Ω·cm^2^. Moreover, while sulfides like Li_3_PS_4_ have an oxidation potential of ∼2.5 V, the oxidation stability of PVDF (∼4.5 V) helps to compensate for this limitation, as shown in Figure 8e [200, 201], expanding the composite electrolyte's working voltage window to 0–4.3 V (vs. Li^+^/Li), making it compatible with high‐voltage cathodes like NMC811.

#### 3.3.3 PVDF‐Based In Situ Polymerized Halide Composite Electrolytes

**FIGURE 9:**
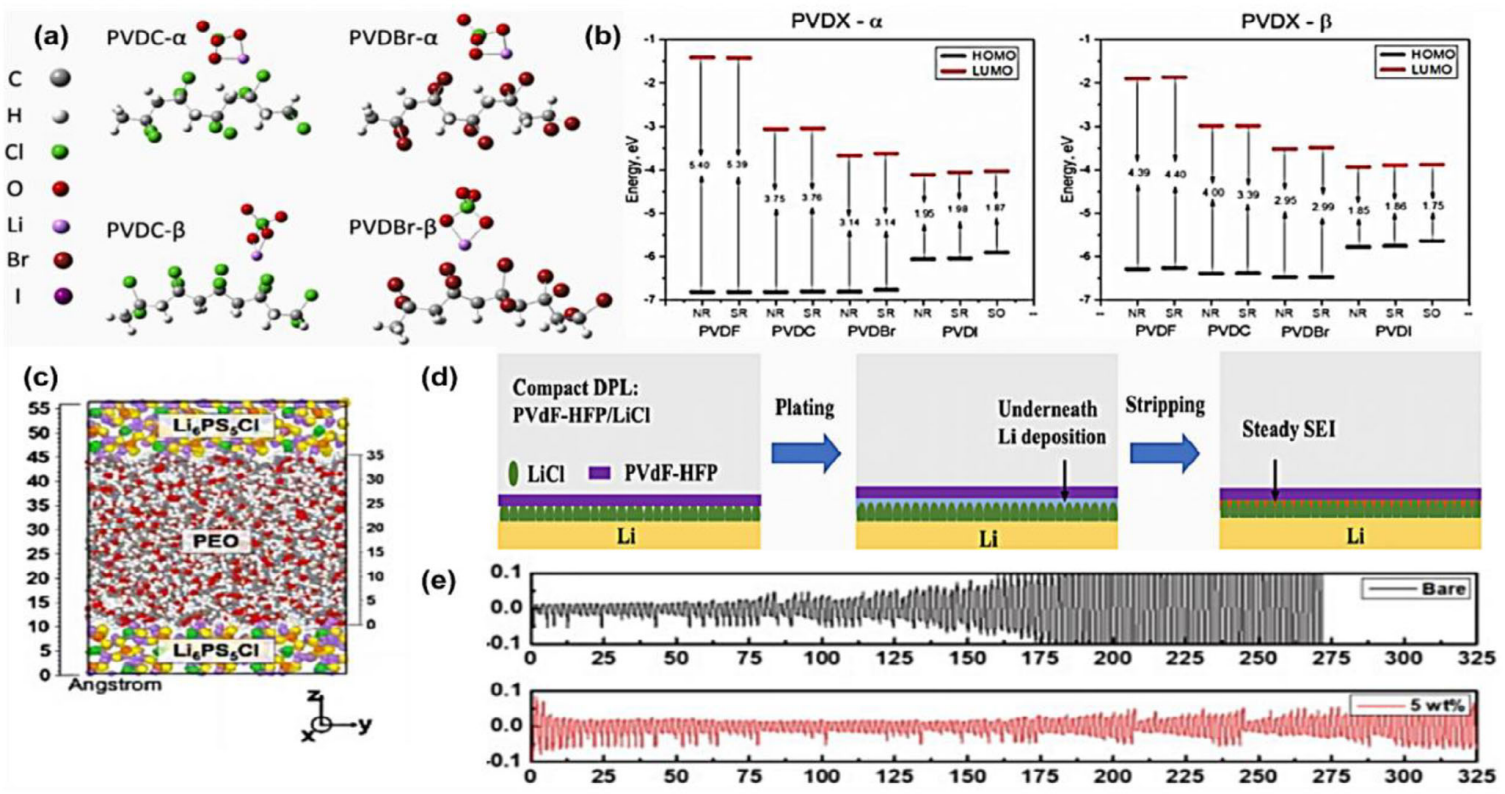
Theoretical and experimental study of polyvinylidene halides and their ion transport properties. (a,b) Theoretical study of polyvinylidene halides. Reproduced with permission from [205]. Copyright 2024, Elsevier.(c) Ion transport at polymer–argyrodite interfaces. Reproduced with permission from [207]. Copyright 2024, American Chemical Society. (d,e) A compositionally favorable and structurally robust dual‐protective layer. Reproduced with permission from [208]. Copyright 2021, Elsevier.

The introduction of halide fillers (e.g., Li_3_YCl_6_, Li_2_ZrCl_6_) into PVDF‐based composite electrolytes enhances ion transport, mechanical strength, and interface stability. The electrostatic interactions between the polar ‐CF_2_ groups in PVDF and halide ions (Cl^−^ or F^−^) facilitate a directional arrangement at the interface, improving adhesion between the polymer and filler and reducing interface defects. These interactions promote uniform dispersion of halide particles, enhancing ion conduction efficiency. Additionally, surface hydroxyl or hydrated layers on halides can form hydrogen bonds with PVDF, further improving the interface stability [203]. The combination of the rigid halide lattice providing high‐conductivity channels and PVDF's amorphous regions enhances ion migration by forming hybrid transport pathways across the halide–polymer interface, enabling ions to migrate through interconnected inorganic domains and the surrounding polymer matrix. The dielectric properties of PVDF can be optimized by controlling the β phase ratio, evidenced in Figure 9a,b [204, 205, 206], which lowers activation energy and further enhances ion transport.

The composite system also benefits from enhanced mechanical properties, as the high modulus of halides like Li_3_InCl_6_ blocks lithium dendrite penetration, while PVDF's flexibility absorbs stress during cycling. This synergistic structure increases the critical current density and improves the stability of the composite electrolyte. Furthermore, as shown in Figure 9c–e [207, 208], the formation of a stable SEI layer on the lithium metal surface, driven by C─F bonds and Cl^−^ ions, helps suppress dendrite growth. Recent studies further show that filler engineering or surface modification can regulate this process. For example, introducing LiF layers, fluorinated coatings, or surface‐modified ceramic fillers can suppress excessive defluorination while promoting the formation of stable LiF‐dominated interphases, thereby reducing parasitic reactions and enhancing long‐term cycling stability of composite electrolytes. Meanwhile, PVDF's hydrophobic nature improves resistance to moisture and hydrolysis, and surface coatings on halide fillers (e.g., Al_2_O_3_ or LiF) further stabilize the interface by mitigating undesired side reactions [209, 210]

### 3.4 PEGMA

**FIGURE 10:**
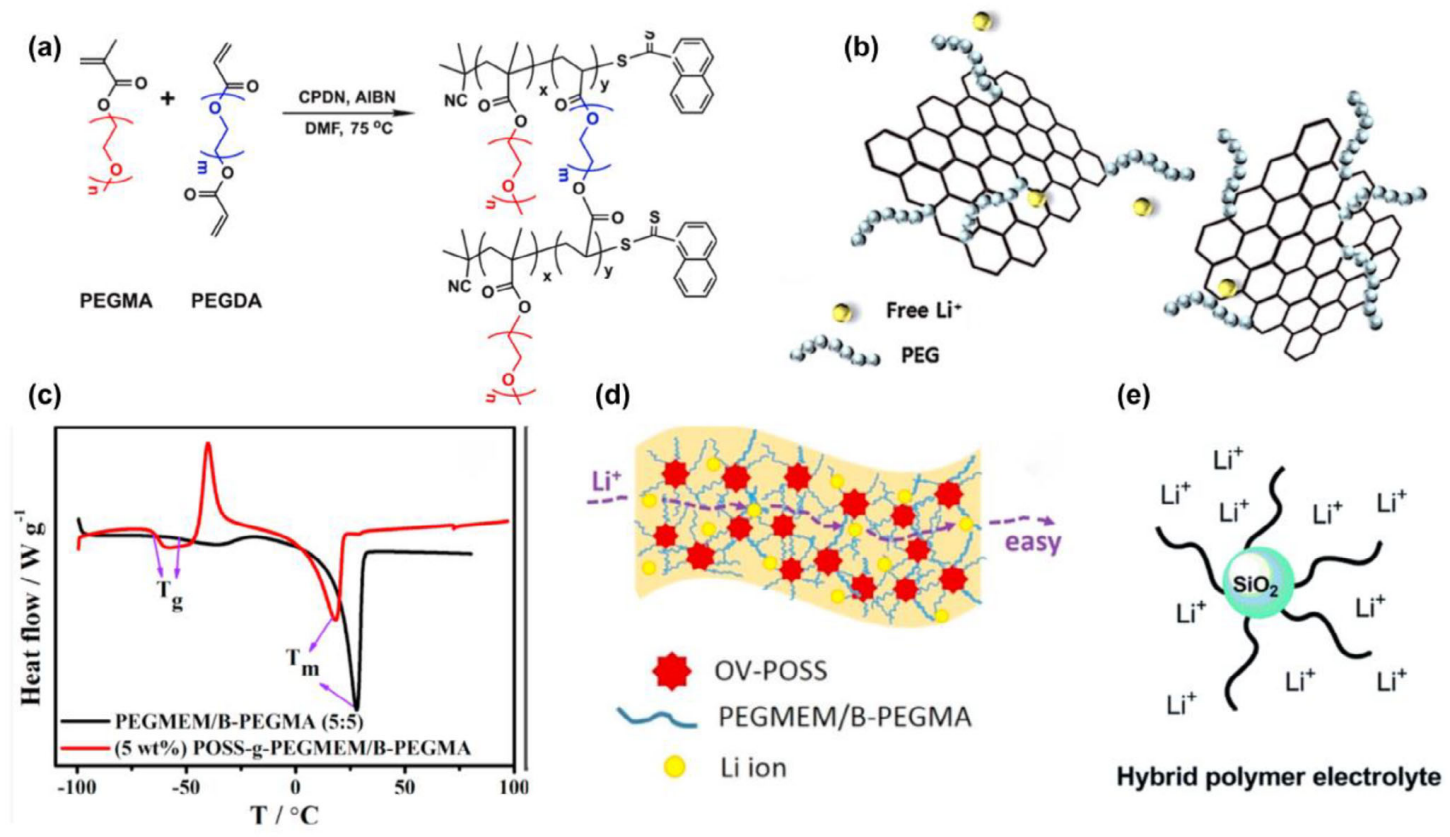
Design and electrochemical properties of PEGMA‐based composite solid‐state electrolytes with modified nanoparticles and graphene oxide. (a) Poly(ethylene oxide)‐based composite polymer electrolytes embedding with ionic bond modified nanoparticles. Reproduced with permission from [211]. Copyright 2019, Elsevier. (b) A novel composite polymer electrolytes containing poly(ethylene glycol)‐grafted graphene oxide. Reproduced with permission [215]. Copyright 2014, Royal Society of Chemistry. (c,d) A star‐shaped solid composite electrolyte containing multifunctional moieties with enhanced electrochemical properties. Reproduced with permission from [216]. Copyright 2018, Elsevier. (e) A composite electrolyte comprised of poly(ethylene oxide) and silica nanoparticles with grafted poly(ethylene oxide)‐containing polymers. Reproduced with permission [221].Copyright 2014, Royal Society of Chemistry.

Poly (ethylene glycol) dimethacrylate (PEGMA) is synthesized from poly (ethylene glycol) (PEG) and methacrylate groups. As described in Figure 10a [211], polymerizable methacrylate end groups form a covalently crosslinked network under ultraviolet (UV) irradiation with a cross‐linking density of 10^−3^ mol cm^−3^ [212]. This structure enhances mechanical strength, increasing the elastic modulus from 0.1 to 10 MPa, and raises the critical current density to 1.5 mA cm^−2^, effectively suppressing lithium dendrite growth. Adjusting monomer concentration enables the preparation of self‐supporting solid electrolyte membranes [213, 214].

As a crucial unsaturated monomer for in situ polymerization, PEG provides several advantages. Its ether oxygens, with lone pair electrons, dynamically coordinate with Li^+^, illustrated in Figure 10b [215], weakening the Coulombic forces between Li^+^ and anions (e.g., TFSI^−^) and enhancing lithium salt dissociation to yield a carrier concentration up to 10^21^ cm^−3^. These oxygen atoms also form Li(EO)_n_^+^ complexes, compressing the solvation shell from 2.5 to 1.8 Å and reducing desolvation energy from 0.45 to 0.28 eV. Meanwhile, flexible chains enable segmental motion, creating dynamic ion channels. Within the crosslinked matrix, local segmental flexibility promotes Li^+^ hopping, described in Figure 10c,d [216], especially when the amorphous content exceeds 70%, yielding superior room‐temperature conductivity compared to systems like PAN discussed before [217]. Additionally, ether oxygens can form hydrogen bonds with cathode hydroxyl groups (e.g., LiFePO_4_), reducing interfacial voids and charge transfer resistance [218]. The hydrogen bonding between PEGMA and plasticizers such as SN imparts self‐healing ability, restoring over 90% of mechanical integrity within 30 min at room temperature, and within 10 min at 60°C.

Meanwhile, there are several challenges for PEGMA‐based in situ polymerized electrolytes. The PEG segments adopt a helical conformation similar to PEO, leading to partial crystallization (30%–40%) at room temperature with limited ion transport for the crystalline regions [219, 220]. Plasticizers (e.g., SN) are typically required to suppress crystallinity. Additionally, high crosslinking density restricts segmental motion, hindering the formation of ion conduction pathways and reducing the dynamic nature of hydrogen bonds and ion–dipole interactions, thereby compromising self‐healing efficiency. Combining PEGMA with inorganic electrolytes effectively mitigates these issues. The ether oxygens in PEGMA side chains act as strong Lewis bases, forming hydrogen bonds and Lewis acid–base interactions with surface hydroxyl groups (–OH) on oxides. These interactions disrupt PEG chain ordering at interfaces, inhibit crystal nucleation, and lower overall crystallinity, enhancing segmental mobility and increasing room‐temperature ionic conductivity (from 10^−6^ to 10^−4^ S cm^−1^). Such interfacial interactions also enhance polymer polarity, promote lithium salt dissociation (e.g., LiTFSI, LiClO_4_), and improve Li^+^ transport. Furthermore, polar groups can adsorb Li^+^ at inorganic interfaces, suppressing anion migration and raising the Li^+^ transference number, thus improving overall ionic performance.

#### 3.4.1 PEGMA‐Based In Situ Polymerized Oxide Composite Electrolytes

Nanoparticles such as Li_7_La_3_Zr_2_O_12_ (LLZO), SiO_2_, Al_2_O_3_, and TiO_2_ possess abundant surface hydroxyl groups and oxygen atoms, which can form hydrogen bonds and Lewis acid–base interactions with the ether oxygen moieties in PEGMA side chains interactions trigger multiple effects at both molecular and mesoscale. The synergy of interfacial coordination, selective Li^+^ transport, mechanical reinforcement, and interphase stabilization makes PEGMA–oxide composites promising for safe and high‐performance solid‐state lithium batteries [222]. Strong interactions between PEGMA and oxides disrupt PEG crystallization, especially at polymer–particle interfaces. Hydrogen bonding between OH groups and ether oxygens inhibits chain packing, increasing the amorphous content from 40%–50% to 70%–80%. Since ionic conduction mainly occurs in amorphous regions with high segmental mobility, as depicted in Figure 10e [221], this enhances the room‐temperature conductivity from 10^−6^ to 10^−4 ^S cm^−1^. Additionally, Oxide nanoparticles can selectively interact with Li^+^ ions. This was further verified by Hu., which demonstrated that the incorporation of SiO_2_ could increase chain mobility, leading to enhanced ionic conductivity. The corresponding CPEs exhibited improved cycling performance and electrochemical stability [211].

The negatively charged oxygen atoms or deprotonated surface groups can electrostatically adsorb Li^+^ while repel bulky anions like TFSI^−^ [221]. This selective adsorption increases the lithium transference number (t^+^) from 0.25 to 0.5, reducing concentration polarization during cycling. Moreover, these interfacial interactions contribute to the stabilization of the electrode–electrolyte interface. The PEGMA–oxide composite layer can form a robust and chemically stable interphase at the lithium metal interface. The polar functional groups within the PEGMA polymer matrix facilitate strong interfacial adhesion, enabling intimate contact between the electrolyte and the lithium surface while suppressing interfacial void formation during cycling. Meanwhile, the incorporation of oxide fillers further regulates the interfacial chemistry. Owing to their strong Lewis basicity, oxide particles can effectively scavenge trace HF and other acidic impurities generated during electrochemical operation, thereby mitigating detrimental side reactions and stabilizing the SEI. In addition, the inorganic oxide framework contributes to the mechanical reinforcement of the composite electrolyte, which helps resist dendrite penetration and maintain structural integrity under repeated lithium plating/stripping. Through the synergistic effects of polymer flexibility and inorganic filler stability, PEGMA–oxide composite electrolytes can effectively enhance interfacial stability and prolong the cycling lifespan of lithium metal batteries.

#### 3.4.2 PEGMA‐Based In Situ Polymerized Sulfide Composite Electrolytes

The ether oxygens in PEGMA act as Lewis bases, dynamically coordinating with Li^+^ at the sulfide–polymer interface, reducing the desolvation barrier and facilitating continuous Li^+^ transfer. This interaction alleviates interfacial charge accumulation, lowering resistance (R_int_) from 300–500 to 50–100 Ω cm^2^. Beyond ionic coordination, PEGMA forms hydrogen bonds with terminal P‐S^−^ and bridging S^2−^ groups in sulfide electrolytes, anchoring polymer chains to the surface. These interactions suppress sulfide particle aggregation, homogenize ion flux, and enhance interfacial adhesion, preventing microvoids and delamination during cycling.

PEGMA also introduces a compliant, cohesive polymeric interphase that accommodates volume changes at the Li‐sulfide interface, buffering mechanical mismatch and suppressing dendrite growth. Its methacrylate groups enable in situ photopolymerization, forming a semi‐interpenetrating network that reinforces mechanical integrity without sacrificing ionic conductivity [223]. Furthermore, PEGMA improves chemical compatibility by encapsulating reactive sulfide surfaces, mitigating the formation of insulating decomposition products like Li_2_S, Li_3_P, and LiCl. The resulting stable, ion‐conductive interphase rich in Li–ether complexes enhances cycling stability and reduces parasitic reactions [223].

#### 3.4.3 PEGMA‐Based In Situ Polymerized Halide Composite Electrolytes

The ether oxygen atoms in PEGMA side chains act as strong Lewis bases coordinating with Li^+^ ions within the halide electrolyte matrix and at the polymer–electrolyte interface. This coordination lowers the desolvation barrier and facilitates continuous ion solvation–desolvation, enhancing lithium‐ion transport. The dynamic formation of Li(EO)_n^+^ alleviates charge accumulation and suppresses space charge layers, reducing interfacial resistance from 150–300 to 50–100 Ω cm^2^ [214, 224]. PEGMA interacts with halide electrolyte surfaces via ion–dipole interactions and hydrogen bonding. Polarizable halide anions (Cl^−^, Br^−^) exhibit favorable electrostatic interactions with the electron‐rich ether groups, stabilizing the polymer–electrolyte interface and preventing interfacial delamination [225]. This anchoring disrupts polymer chain crystallization near halide particles, increasing local amorphous content and boosting interfacial Li^+^ mobility.

PEGMA forms a soft, flexible interlayer that accommodates interfacial stress and suppresses microcracks during cycling. Its photopolymerizable methacrylate groups enable in situ crosslinked networks, improving dimensional and mechanical stability without sacrificing ionic mobility. This dual reinforcement elevates the critical current density for dendrite suppression to above 0.5 mA cm^−2^ [226]. Beyond mechanical reinforcement, PEGMA also contributes to interfacial chemical stability. PEGMA mitigates moisture‐induced degradation by physically encapsulating halide particles, shielding them from hydrolysis and stabilizing the interface through hydrogen bonding and ion–dipole interactions. The PEGMA matrix also restricts anion mobility via steric hindrance and preferential Li^+^ solvation, increasing the lithium‐ion transference number in the composite electrolyte.

## 4 Cyclic Monomers

Cyclic monomers, particularly cyclic ethers like ethylene oxide (EO) and propylene oxide (PO)—are promising building blocks for high‐performance solid‐state polymer electrolytes due to their unique structural and functional properties. Structurally, these monomers feature closed‐ring frameworks containing polar groups (e.g., C–O–C) that strongly coordinate with Li^+^ ions. Under catalytic conditions, they undergo ring‐opening polymerization to form linear or crosslinked polymers such as poly (ethylene oxide) (PEO).

A key advantage of cyclic monomers is their ability to reduce crystallinity and increase the amorphous phase in the resulting polymer, enhancing chain mobility and creating efficient ion transport pathway across polymer‐inorganic interfaces. The inherent structural irregularity and minimal side groups suppress crystallization, while heteroatoms like ether oxygen improve chain flexibility, promoting segmental motion essential for ion conduction. Polymers derived from cyclic monomers also offer excellent chemical stability. Their saturated backbones resist chain scission and crosslinking under thermal or electrochemical stress, which is crucial for stability against reactive electrodes like lithium metal. Additionally, abundant polar groups enhance interfacial compatibility with inorganic fillers through Lewis acid–base interactions, stabilizing the interface and promoting uniform filler dispersion.

Overall, cyclic monomer‐based polymers combine molecular‐level design flexibility with robust physicochemical properties, making them highly attractive for next‐generation solid‐state electrolytes [227]. Their synergistic effects—reducing crystallinity, enhancing Li^+^ solvation and mobility, and improving compatibility with inorganic fillers—highlight their growing relevance in advanced energy storage research.

### 4.1 PEO

PEO is a type of polyether obtained by the ring‐opening polymerization of ethylene oxide (epoxide rings) [228, 229]. Its structure consists of repeating ‐OCH_2_CH_2_‐ units, forming a long‐chain polymer in which ethylene (‐CH_2_CH_2_‐) and oxygen (‐O‐) groups alternate. The molecular formula (C_2_H_4_O)*_n_* indicates that the polymer's molecular weight depends on the number of repeating units. The ethylene segments form the backbone of the polymer chain, and the presence of oxygen‐carbon bonds imparts significant flexibility within a given temperature range. This flexibility enables rotational and oscillatory motion of polymer segments, which facilitates Li^+^ migration through hybrid transport pathways spanning the polymer matrix and neighboring inorganic electrolyte particles. Through these interfacial relay pathways, Li ions can migrate continuously across polymer‐inorganic boundaries, thereby enhancing the overall ionic conductivity of the composite electrolyte [230]. Another crucial factor is the high polarity of the oxygen atoms in PEO molecules. Oxygen atoms possess lone electron pairs and act as Lewis bases, while lithium ions, as Lewis acids, coordinate with these electrons to form stable complexes. This coordination facilitates the solvation of Li^+^ in solid‐state electrolytes, reducing the energy barrier for ion migration, stabilizing Li^+^, and enhancing ion transport [113]. As a result, the charge carrier concentration can reach 10^20^–10^21^ cm^−3^ [231].

The mechanical properties of PEO, which significantly impact its electrochemical performance, are strongly dependent on molecular weight. Longer PEO chains provide greater mechanical strength. Additionally, the intertwining of long polymer chains leads to the formation of a stable 3D network, enhancing the mechanical robustness of the polymer membrane. However, higher molecular weight also results in more ordered chain packing, increasing crystallinity and thereby limiting ion transport. In contrast, shorter PEO chains exhibit more pronounced end‐group effects and greater segmental mobility, leading to lower crystallinity and a reduced glass transition temperature (Tg). This enhances lithium‐ion transport but may compromise mechanical stability at elevated temperatures or during prolonged operation. Hence, careful control of polymerization conditions is essential to balance PEO's crystallinity, flexibility, ionic conductivity, and mechanical properties [232]. During in situ polymerization with inorganic electrolyte materials, the segmental structure and crystallinity of PEO are effectively modified. Inorganic particles interact with PEO chains via van der Waals forces, hydrogen bonding, or ionic interactions, reducing PEO's crystallinity and thereby enhancing ionic conductivity. The addition of PEO in inorganic electrolyte can also increase the ion transport channel, improve the mechanical properties and interface.

#### 4.1.1 PEO‐Based In Situ Polymerized Oxide Composite Electrolytes

**FIGURE 11:**
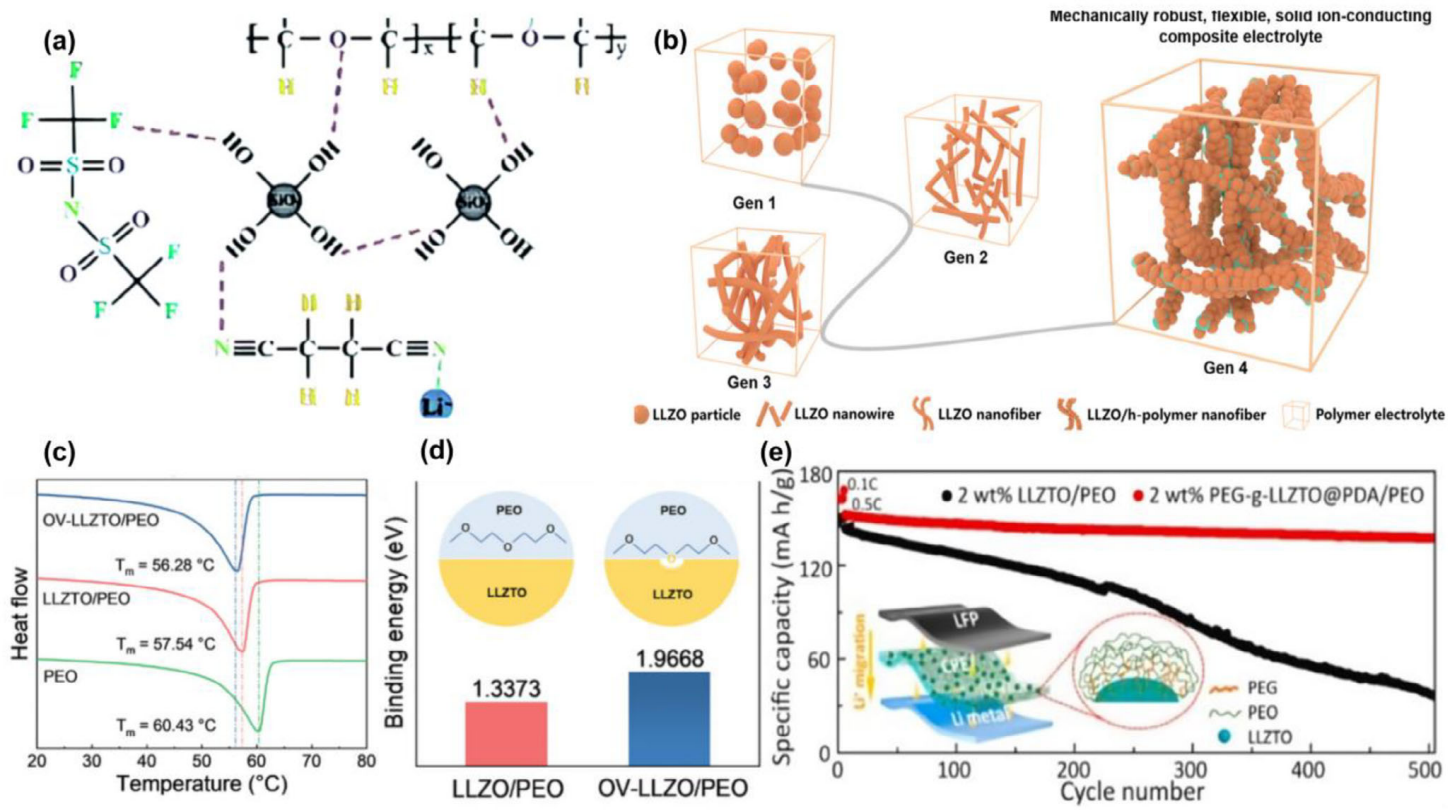
Functional PEO/oxide composite electrolytes with enhanced interfacial compatibility and mechanical stability. (a) Hydrogen bonding enhanced SiO_2_/PEO composite electrolytes. Reproduced with permission from [233]. Copyright 2022, Royal Society of Chemistry. (b) Surface defects reinforced polymer‐ceramic interfacial anchoring. Reproduced with permission from [234]. Copyright 2023, Wiley. (c) A multifunctional Janus layer for LLZTO/PEO composite electrolyte. Reproduced with permission from [235]. Copyright 2024, Elsevier. (d) PEG brush on LLZTO toward intimate interfacial compatibility. Reproduced with permission from [236]. Copyright 2021, Elsevier. (e) Electrolyte membrane with conducting oxide‐enhanced 3D nanofiber networks. Reproduced with permission from [237]. Copyright 2021, American Chemical Society.

PEO polymerization similarly improves the mechanical properties and interfacial stability of oxides. For example, the ether oxygen in PEO chains forms hydrogen bonds with hydroxyl groups on the oxide surface, displayed in Figure 11a [233], strengthening the interfacial adhesion between the filler and the matrix. This suppresses crack propagation and improves mechanical strength, increasing the elastic modulus from 0.1 GPa (pure PEO) to 1–3 GPa in the composite. Additionally, oxide nanoparticles adsorb PEO chains via van der Waals interactions, reducing filler aggregation and polymer chain entanglement. With the assistance of hydrogen bonding and Lewis acid‐base interactions, the in situ polymerization enables uniform filler dispersion. The terminal hydroxyl groups of PEO condense with active sites on the oxide surface to form covalent bonds (Si‐O‐C), further strengthening interfacial adhesion. This reduces interfacial resistance from >1000 Ω·cm^2^ (pure PEO) to <100 Ω·cm^2^, enhancing cycling stability.

Similar to sulfides, the polymerization of PEO with oxides also facilitates ion transport. In addition to the conventional Li^+^ transport pathway dominated by dipole interactions between Li^+^ and PEO ether oxygen, Li^+^ can also migrate via coordinated hopping along surface oxygen sites of oxides [106, 238], such as lattice oxygen in LLZO demonstrated in Figure 11b [102, 234]. This mechanism lowers the activation energy from 1.0 eV (pure PEO) to 0.5–0.7 eV and increases the room‐temperature ionic conductivity from 10^−7^ S cm^−1^ (pure PEO) to 10^−4^ S cm^−1^ in LLZO‐based composites. On the other hand, oxides contribute additional benefits to PEO polymerization, as shown in Figure 11c [235]. Their surface Lewis acidic sites (e.g., Al^3+^, Ti^4+^) adsorb anions (TFSI^−^), facilitating Li^+^ dissociation through charge shielding while inhibiting anion migration, thereby increasing free Li^+^concentration (from 10^−3^ to 10^−2^ m). Furthermore, as illustrated in Figure 11d,e [236, 237, 239], rigid oxide particles (e.g., ZrO_2_) serve as physical crosslinkers, limiting PEO chain motion, disrupting crystallinity, and stabilizing the amorphous phase to enhance ionic conductivity. However, oxide rigidity hinders uniform nanoparticle dispersion via traditional mechanical blending, leading to pore defects. Additionally, PEO's low melting point causes structural collapse at high temperatures, severely reducing ionic conductivity and increasing side reactions with lithium metal.

#### 4.1.2 PEO‐Based In Situ Polymerized Sulfide Composite Electrolytes

When sulfide nanoparticles are introduced into PEO during in situ polymerization, they act as effective heterogeneous nucleation sites due to their substantial interfacial free energy difference and favorable wettability with the polymer matrix. This interfacial interaction disrupts long‐range crystalline ordering and substantially increases the amorphous fraction of PEO, thereby enhancing segmental mobility and shortening chain relaxation times. The resulting increase in polymer dynamics facilitates Li^+^ coordination exchange and contributes to improved ionic transport [240].

**FIGURE 12:**
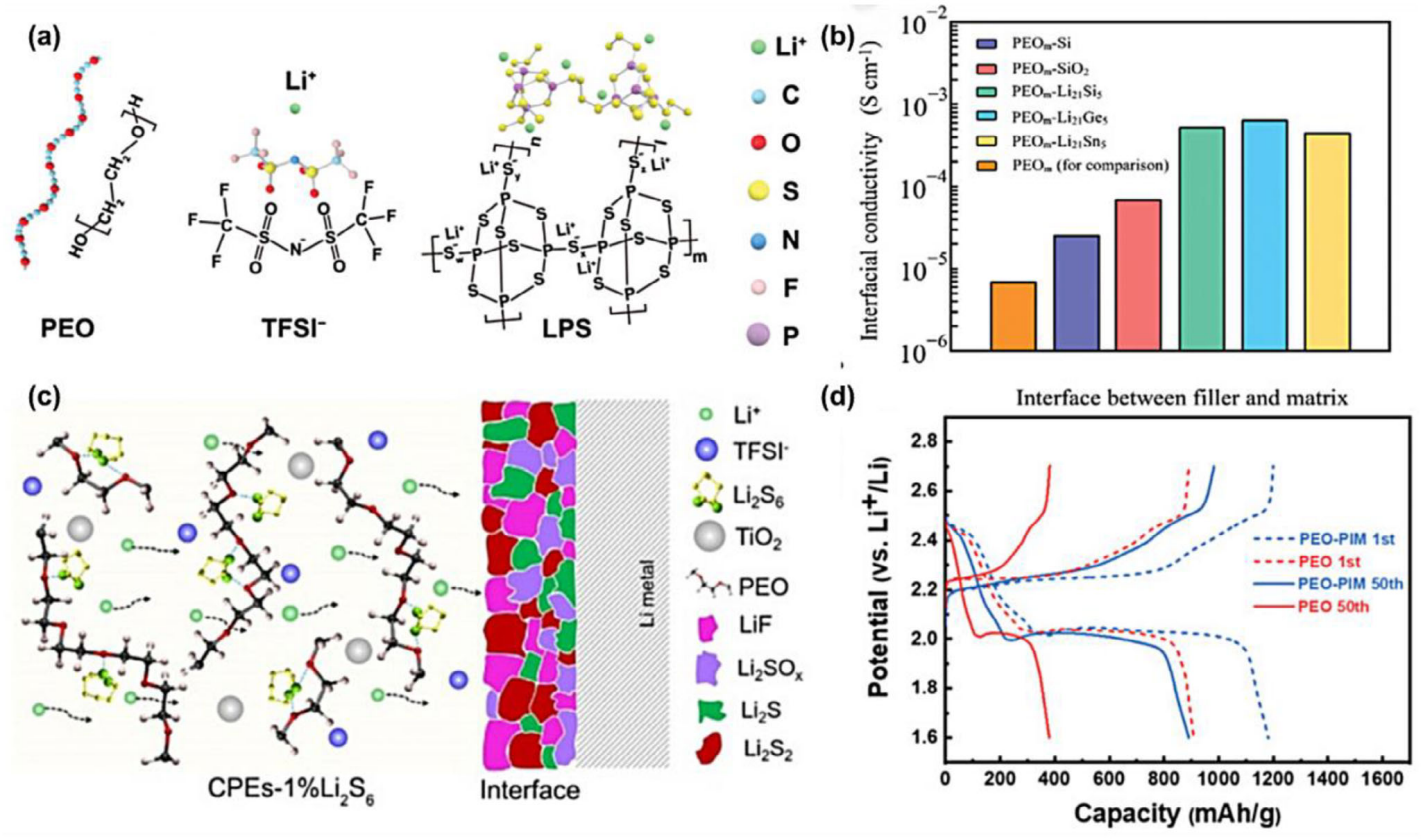
Multifunctional PEO‐based polymer electrolytes with integrated SEI layers and enhanced interfacial compatibility. (a) In situ formation of Li_3_PS_4_ in the SEI Layer; [241] (b) A Li‐Rich Artificial SEI Layer. Reproduced with permission from [245]. Copyright 2022, Wiley. (c) A Li_2_S_6_ integrated PEO‐based polymer electrolyte. Reproduced with permission from [246]. Copyright 2021, Wiley. (d) A multifunctional framework of PIM‐1. Reproduced with permission from [247]. Copyright 2021, Wiley.

At the molecular level, Li^+^ serve as dynamic bridges between the ether oxygen atoms of PEO and sulfide surface species. Surface‐exposed S^2−^ or PS_4_^3−^units, particularly at defect or termination sites, may weakly interact with Li^+^, forming a three‐component coordination environment. Rather than constituting strong chemical bonding, these interactions are better understood as interfacial electrostatic modulation that alters local ion distribution. The negatively charged sulfide domains generate localized electric fields that polarize PEO dipoles and promote Li^+^ enrichment near the interface, consistent with space‐charge redistribution concepts. This interfacial field weakens Li^+–^anion pairing and increases the effective population of dissociated Li^+^ species while simultaneously imposing a higher energetic penalty for bulky anion approach. The resulting partial decoupling of cation and anion transport improves ionic conductivity and Li^+^ transference characteristics, as displayed in Figure 12a,b [241, 242].

Incorporating PEO into sulfide‐based electrolytes not only disrupts crystallinity but also introduces additional Li^+^ transport pathways. For instance, the rigid lattice of Li_10_GeP_2_S_12_ serves as a high‐conductivity Li^+^ transport channel. Through a ‘segment‐ion cooperative migration’ mechanism—akin to ion hopping—PEO chain segments bridge adjacent lattice pathway. This synergistic effect lowers the activation energy (Ea) from 0.2 eV (pure sulfide) to 0.15 eV and enhances ionic conductivity to >10^−3^ S cm^−1^ [243]. Zhao's group systematically verified that the incorporation of LGPS into PEO positively influences the overall ionic conductivity and electrochemical stability [244].

In addition, the in situ polymerization of sulfide electrolytes with PEO enhances both mechanical and interfacial stability. The combination of PEO's flexibility (elongation at break >300%) with the high modulus of sulfides (e.g., ∼20 GPa for Li_3_PS_4_) results in a composite structure. The rigid sulfide particles act as barriers to lithium dendrite penetration, while PEO's elastic deformation helps absorb the cyclic stresses at the interface. At the interface level, trace water adsorbed on the sulfide surface forms hydrogen bonds with PEO's ether oxygen groups, effectively blocking further side reactions with water. The ether oxygen groups are also preferentially oxidized on the high‐voltage cathode side, as shown in Figure 12c,d [245, 246, 247], creating a polyether‐based passivation layer that prevents further sulfide decomposition, raising the oxidation potential of the composite system from 2.5 to 4.0 V (vs Li^+^/Li) [248].

Nevertheless, these advantages remain temperature‐dependent. PEO softening near its melting transition limits mechanical robustness at elevated temperatures, while sulfide phases may undergo structural or chemical instability under extreme thermal conditions. Thus, the performance gains observed in PEO‐sulfide systems arise from interfacial electrostatic regulation, dynamic polymer‐assisted transport, and mechanical complementarity operating within a constrained thermomechanical window.

#### 4.1.3 PEO‐Based In Situ Polymerized Halide Composite Electrolytes

**FIGURE 13:**
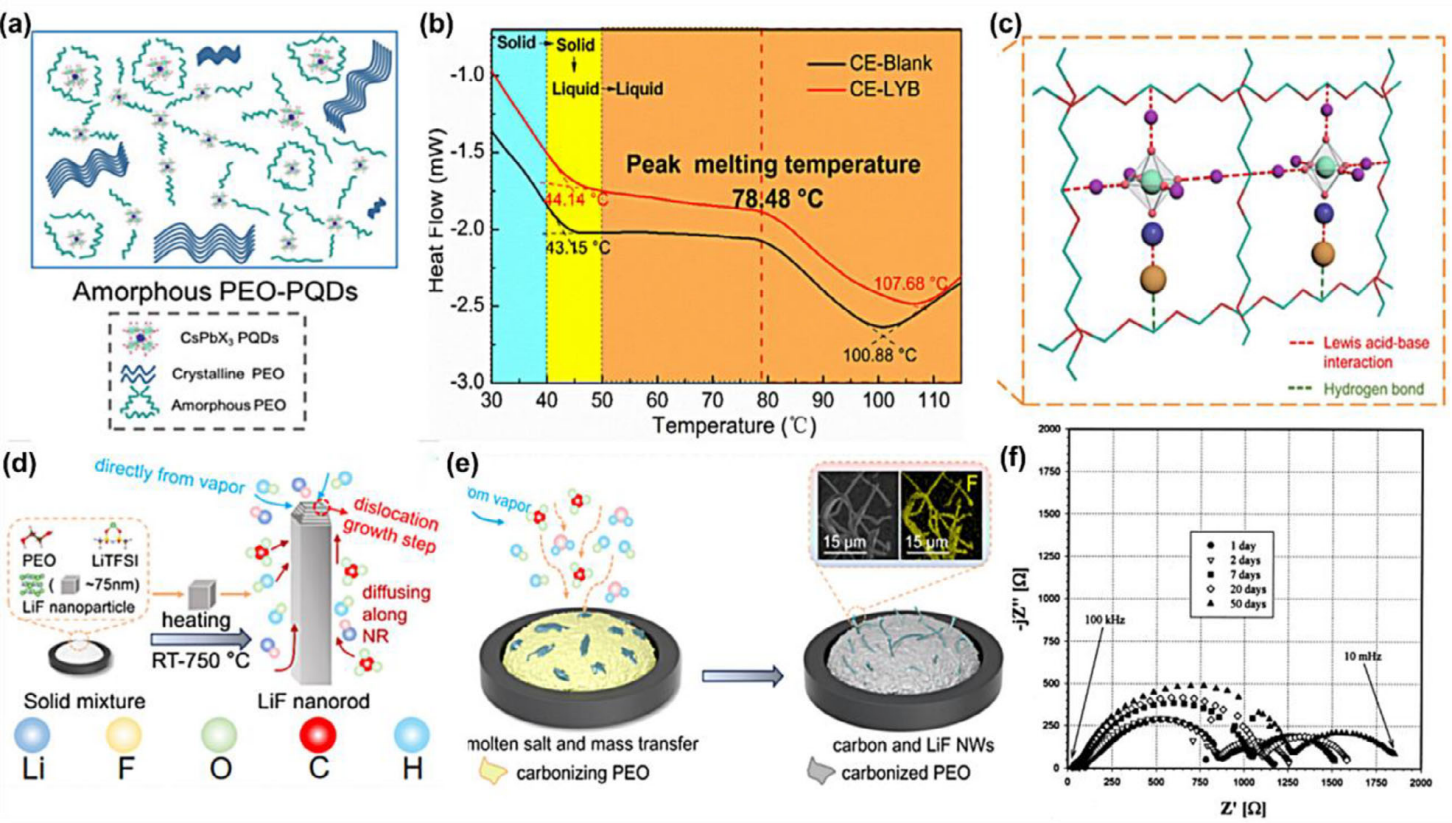
Interfacial engineering and thermal properties of organic–inorganic hybrid PEO‐based composite electrolytes. (a) An organic–inorganic hybrid perovskite/polymer composite thin film. Reproduced with permission from [254]. Copyright 2017, Wiley. (b) A tri‐salt CPE with temperature switch function. Reproduced with permission from [249]. Copyright 2024, Wiley. (c,d) Perovskite quantum dots for lewis acid–base interactions and interface engineering. Reproduced with permission from [250]. Copyright 2021, American Chemical Society. (e) Upcycling spent PEO electrolytes into lithium fluoride nanowhiskers. Reproduced with permission from [255]. Copyright 2025, American Chemical Society. (f) A PEO‐LiX composite electrolyte to enhance interfacial properties. Reproduced with permission from [256]. Copyright 2000, Elsevier.

The combination of PEO with halides exhibits advantages similar to those observed with oxides and sulfides. Halide nanoparticles (e.g., Li_3_InCl_6_, <50 nm) serve as nucleation sites, disrupting PEO chain ordering and lowering crystallinity, as evidenced in Figure 13a,b [249, 250]. This increased amorphous content enhances chain mobility, boosting room‐temperature ionic conductivity from 10^−7^ to 10^−5^ S cm^−1^ [251]. Moreover, spatially confined PEO chains on halide surfaces form dense amorphous regions, where cooperative segmental motion accelerates Li^+^ hopping, reducing activation energy to 0.18 eV.

Similar to previous discussions, mechanically, the combination of high‐modulus halides (∼25 GPa, e.g., Li_3_YCl_6_) and flexible PEO (∼1 MPa) establishes a gradient modulus structure. While the halide layer resists dendrite penetration, the PEO layer accommodates volume fluctuations via viscoelastic deformation, increasing the critical current density from 0.2 to 1.5 mA cm^−2^ [252]. At the interface, halides with a higher oxidation potential (∼4.5 V vs Li^+^/Li) than PEO (∼4.0 V) promote a LiCl‐Li_2_O passivation layer on high‐voltage cathodes (e.g., NMC811), preventing PEO degradation. On the lithium metal side, ether oxygen in PEO reacts with Li, forming an SEI enriched with Li_2_O, while Cl^−^ from halides contributes to LiCl formation. This synergistic SEI structure enhances ionic transport and mechanical resilience [253].

Furthermore, PEO polymerization with halides introduces distinctive benefits. As illustrated in Figure 13c,d [254], Li^+^ can coordinate with both the ether oxygen in PEO and Cl^−^ within the halide lattice, forming a stable Li‐O‐Cl coordination network. This structure not only strengthens interfacial adhesion but also lowers the activation energy for Li^+^ transport (from 0.4 to 0.25 eV). Additionally, the lone‐pair electrons of PEO's oxygen groups (‐O‐) interact with halides like Li_3_YCl_6_ via weak hydrogen bonding or dipole‐dipole interactions with Cl^−^ or F^−^. For example, Cl^−^ (electronegativity 3.0) can electrostatically attract PEO's ether oxygen lone‐pair electrons, creating dynamic bonding that reduces interfacial defects and significantly lowers interfacial resistance (from >1000 to <300 Ω·cm^2^, as shown in Figure 13e,f [255, 256]. However, due to the intrinsic properties of PEO, the electrolyte formed by PEO polymerization with halides still suffers from elastic failure at elevated temperatures and restricted chain segment mobility at room temperature.

### 4.2 PDOL

PDOL is a copolymer or graft polymer derived from polyethylene oxide (PEO) and Dimethylolpropane (DMP) [257, 258]. Its structure comprises long‐chain PEO segments alternated or grafted with DMP units, represented by the molecular formula ‐CH_2_‐CH‐O‐_n_‐C(CH_3_)_2_CH_2_OH. The DMP portion contains two hydroxyl groups (‐OH), which typically act as grafting sites or copolymer units, linking with the PEO chain. While the above structural description provides the basic chemical framework of PDOL‐derived polymers, the purpose of this section is not merely to catalog polymer chemistries. Instead, the focus is to analyze how different cyclic ether–derived monomers influence the formation mechanisms and interfacial behavior of in situ polymerized composite electrolytes. In this context, distinguishing between PEO‐ and PDOL‐based systems is particularly important, as these polymers regulate network formation, polymerization pathways, and polymer–inorganic interfacial evolution in fundamentally different ways.

The hydroxyl groups can interact with other ions or molecules, enhancing the electrolyte's tunability [259]. The hydrophilicity of PDOL aids in adjusting the solubility of composite materials, which, when combined with other fillers (e.g., oxides, sulfides), can further improve the electrolyte's performance. Additionally, the structure contains an acrylate group substituted with two methyl (‐CH_3_) groups, providing PDOL with a balance of flexibility and rigidity [260]. PDOL demonstrates excellent chemical stability due to the absence of highly reactive groups [261]. PDOL exhibits a relatively wide electrochemical stability window, particularly under high‐voltage operation, which is primarily attributed to the absence of readily oxidizable functional groups within its molecular structure [262]. This enhances its chemical stability and prevents decomposition, ensuring reliable performance over long‐term use in solid‐state batteries. The stable C─O and C─C bonds within PDOL contribute further to its robust chemical properties [263].

Despite these advantages, PDOL has certain limitations as a solid electrolyte [264]. The presence of hydroxyl groups (‐OH) renders it susceptible to moisture absorption, leading to changes in its physical properties, such as reduced ion conductivity and increased brittleness. Furthermore, PDOL's ion migration number is relatively low [265]. When the PDOL molecular chains coordinate with cations, ion pairs or clusters can form, and the electrostatic interactions between anions and cations reduce the migration of cations. Additionally, as cations migrate between ether‐oxygen linkages, they must overcome energy barriers, and the structural changes during coordination also affect ion migration rates. This complexity prevents cations from migrating independently, as they are influenced by both anions and the coordination structure, further reducing the ion migration number.

#### 4.2.1 PDOL‐Based In Situ Polymerized Oxide Composite Electrolytes

**FIGURE 14:**
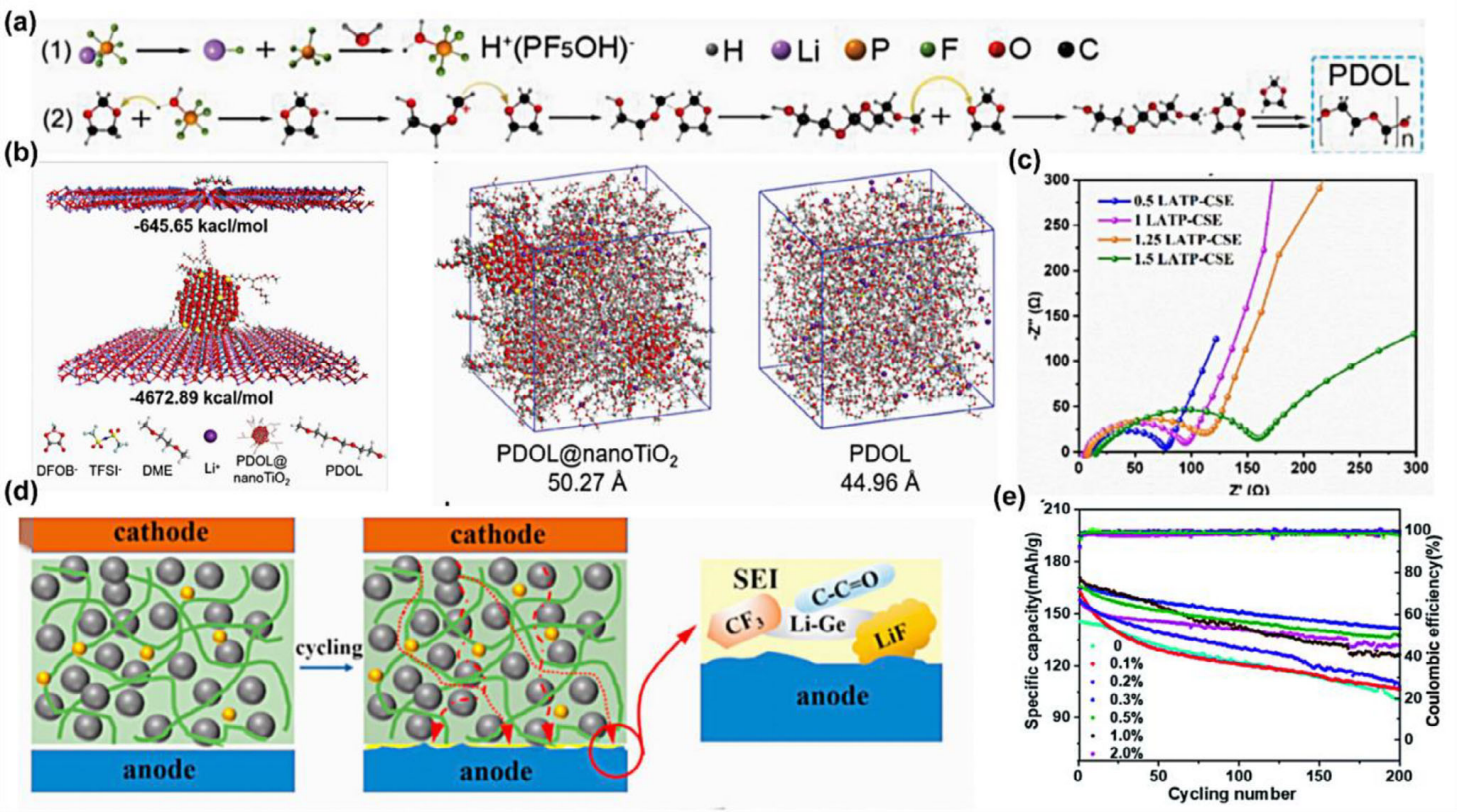
Advanced PDOL‐based composite electrolytes with nanoparticle crosslinking and enhanced ionic conductivity for solid‐state battery applications. (a) In situ catalytic polymerization of a highly homogeneous PDOL composite electrolyte. Reproduced with permission from [269]. Copyright 2022, Wiley. (b) In situ hybrid crosslinking polymerization of nanoparticles. Reproduced with permission from [39]. Copyright 2024, Wiley. (c) A gradient PDOL/LLZTO composite electrolyte. Reproduced with permission from [268]. Copyright 2022, American Chemical Society. (d) A high ionic conductive PDOL/LAGP composite solid electrolyte film. Reproduced with permission from [271]. Copyright 2023, Elsevier. (e) Thin LATP‐based composite solid electrolyte with a reinforced interface of in situ formed poly(1,3‐dioxolane). Reproduced with permission from [272]. Copyright 2023, Elsevier.

The oxygen atoms in PDOL are capable of forming hydrogen bonds with hydroxyl groups on the surface of oxides. Such hydrogen bonding enhances interfacial adhesion, thereby facilitating lithium‐ion transport and improving ionic conductivity at the interface [266]. Additionally, PDOL interacts electrostatically with oxide surfaces. These interactions promote uniform PDOL coating on oxide surfaces, increasing interfacial contact area and facilitating the formation of continuous lithium‐ion conduction pathways [267]. Furthermore, as illustrated in Figure 14a,b [39, 268, 269], electrostatic interactions modulate lithium‐ion distribution and migration within the composite electrolyte, promoting preferential migration along the PDOL–oxide interface and thereby enhancing migration efficiency [266, 270]. PDOL possesses electron‐donating functionalities, enabling coordination with lithium ions and facilitating their solvation, as shown in Figure 14c. Inspired by this, Li et al. proposed a multifunctional gradient solid‐state electrolyte, which possesses a high ioinc conductivity of 2.9 × 10^−4^ S cm^−1^with a wide electrochemical window up to 4.9 V vs. Li^+^/Li [268]. When compounded with oxides, their presence alters the solvation structure and local environment of lithium ions in PDOL. The surface charge distribution and lattice structure of oxides influence the coordination configuration and binding strength between PDOL and lithium ions, as demonstrated in Figure 14d,e [271, 272, 273], potentially weakening coordination interactions [274]. This modification renders the lithium‐ion solvation shell more labile, thereby facilitating ion dissociation and migration, which is essential for improving ionic conductivity in solid‐state battery systems [275, 276].

#### 4.2.2 PDOL‐Based In Situ Polymerized Sulfide Composite Electrolytes

The PDOL‐sulfide composite electrolyte exhibits significant improvements in ionic conductivity, interface stability, mechanical strength, and chemical stability through synergistic effects between PDOL and sulfides like Li_6_PS_5_Cl. Highly conductive sulfides construct a rigid framework within the flexible PDOL matrix, establishing a continuous ionic conduction network [277]. This architecture facilitates lithium‐ion transport by providing uninterrupted conduction pathways and supplementary coordination sites via the ether‐oxygen functionalities in PDOL, thereby reducing the activation energy for ion migration and enhancing overall ionic transport kinetics. Furthermore, interfacial interactions between sulfide surfaces and PDOL disrupt the polymer's ordered structure, increasing the amorphous phase fraction and reducing the glass transition temperature (T_g_), thereby significantly enhancing the composite electrolyte's low‐temperature performance [278].

The PDOL interlayer, functioning as a flexible and adaptive interface, mitigates direct contact between sulfide electrolytes and lithium metal, promoting the formation of a stable solid electrolyte interphase (SEI) predominantly composed of Li_2_O and Li‐OR species, while effectively suppressing the generation of high‐resistance byproducts such as Li_2_S. This synergistic interfacial stabilization reduces charge transfer resistance and markedly enhances the cycling stability of Li|composite electrolyte|Li symmetric cells. Additionally, PDOL contributes to cathode passivation by forming a compact polymeric interphase on the high‐voltage cathode surface, thereby inhibiting sulfide decomposition and oxidation reactions, while scavenging reactive O^2−^ radicals. These effects collectively broaden the electrochemical stability window and improve interfacial compatibility between the cathode and electrolyte [279].

The composite electrolyte also shows enhanced mechanical properties due to the high modulus of sulfides and the low modulus of PDOL, creating a ‘rigid‐flexible’ structure that disperses external stress and absorbs deformation energy. This structure inhibits dendrite penetration and increases compressive strength. Moreover, the viscoelastic characteristics of PDOL, coupled with the rigid sulfide framework, afford efficient stress buffering during electrode volume fluctuations, thereby further enhancing the mechanical integrity and long‐term structural stability of the electrolyte [280]. Chemical stability is further improved by the weak coordination bonds formed between the ether‐oxygen groups in PDOL and the S^2−^ in sulfides, reducing side reactions with electrolyte solvents and minimizing byproduct formation. PDOL's hydrophobic nature also prevents moisture intrusion, enhancing storage stability. The presence of sulfide particles as physical crosslinking points limits PDOL's thermal motion, while PDOL's ether‐oxygen chains capture free radicals generated during sulfide decomposition, improving thermal stability.

Nevertheless, several limitations persist. Surface sulfur anions and high‐valent sulfur species present in sulfides can nucleophilically attack the ether‐oxygen linkages within PDOL, resulting in polymer chain scission or undesired crosslinking, which generates interfacial byproducts and elevates interfacial resistance. The fabrication of such composites is further challenged by the inherent sensitivity of sulfide precursors to moisture and oxygen, alongside the strict processing parameters necessary for effective PDOL curing. Furthermore, the poor interfacial wettability between sulfides and PDOL impedes the construction of continuous 3D ion conduction pathways, necessitating the addition of substantial plasticizer content, which consequently deteriorates the electrolyte's thermal stability and dendrite suppression performance.

#### 4.2.3 PDOL‐Based In Situ Polymerized Halide Composite Electrolytes

**FIGURE 15:**
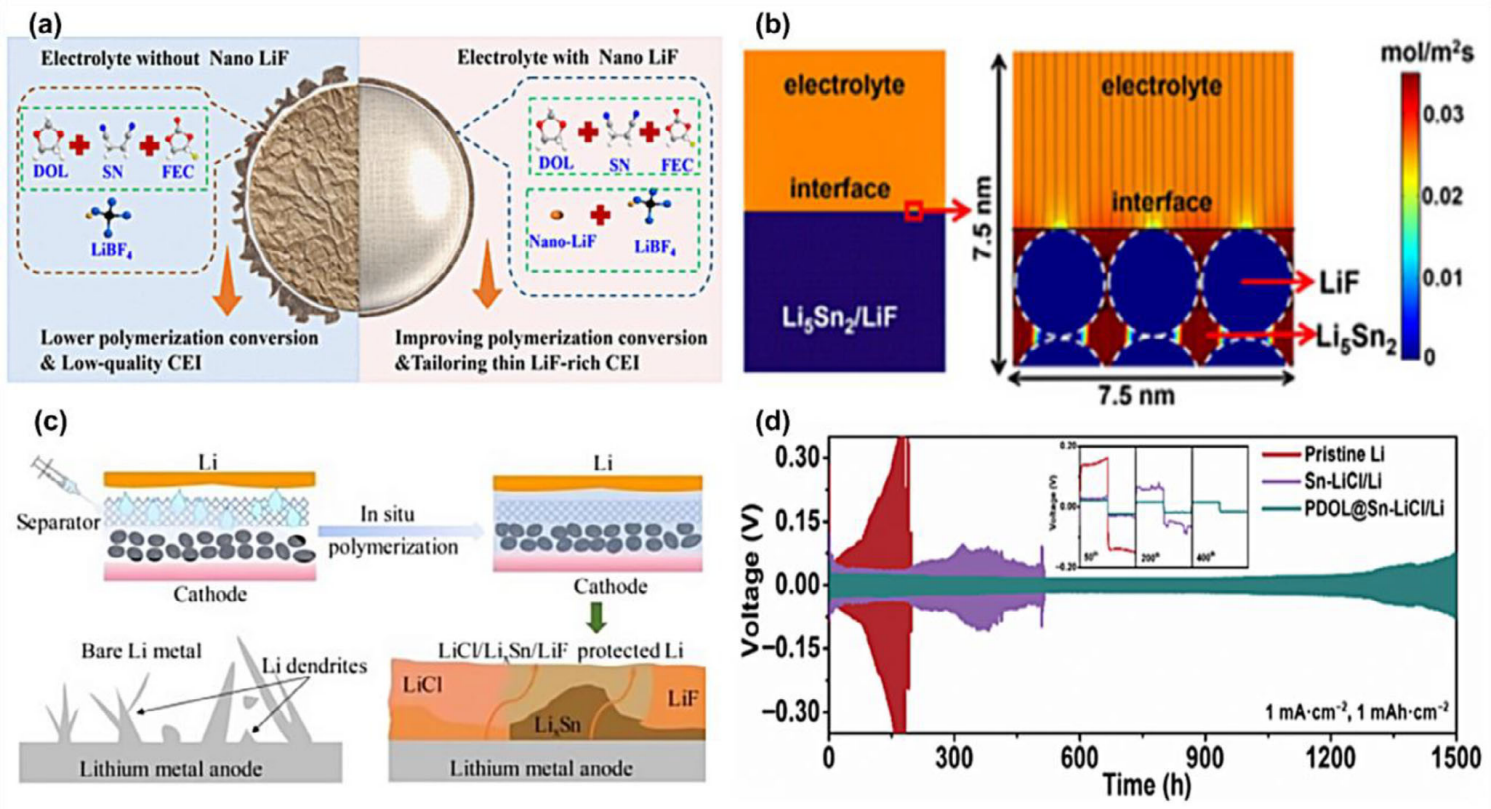
In situ polymerization and LiF‐catalyzed interface engineering in PDOL‐halide based electrolytes. (a) Nano LiF‐enabled in situ synergistic catalytic polymeric electrolytes. Reproduced with permission from [281]. Copyright 2025, American Chemical Society. (b) SnF_2_‐catalyzed formation of polymerized dioxolane as solid electrolyte. Reproduced with permission from [282]. Copyright 2021, Wiley. (c) SnCl_4_ initiated formation of polymerized solid polymer electrolytes. Reproduced with permission from [284]. Copyright 2024, Elsevier. (d) In situ constructed polymer/alloy composite as an artificial solid electrolyte interphase. Reproduced with permission from [283]. Copyright 2023, Springer Nature.

PDOL infiltrates the grain boundaries of halide particles, forming a flexible filler layer that reduces the resistance to lithium‐ion transport at these boundaries. As displayed in Figure 15a,b [281, 282], the ether‐oxygen groups in PDOL interact with the chloride ions on the halide surfaces, improving wettability and facilitating efficient ion conduction. The combined movement of PDOL and halides lowers the activation energy for lithium‐ion migration between the polymer phase and halide electrolyte particles, enhancing ion transport efficiency [281]. PDOL also improves interface stability by forming a flexible protective layer between the halides and lithium metal, preferentially reacting with lithium to generate a stable SEI film that prevents unwanted reactions between halides and lithium, shown in Figure 15c,d [283, 284, 285]. Additionally, PDOL helps form a dense polymer layer on the surface of high‐voltage cathodes (e.g., NCM811), inhibiting halide oxidation at high voltage and reducing oxygen‐related side reactions at the cathode interface [286]. In terms of mechanical properties, halides like Li_3_InCl_6_, with their high modulus, serve as rigid support points, maintaining the structural integrity of the electrolyte and inhibiting lithium dendrite penetration. PDOL's viscoelasticity absorbs deformation energy during cycling, reducing stress at dendrite tips and enhancing the electrolyte's mechanical strength [287, 288].

Although the bulk transport mechanisms of PEO and PDOL are similar, their behavior during in situ polymerization differs in several aspects. PEO‐based networks are often formed through the polymerization of EO‐containing oligomers or crosslinkable precursors, where the degree of crosslinking critically influences crystallinity suppression and interfacial adhesion. PDOL systems, on the other hand, are frequently polymerized directly within the electrode framework via cationic initiators, enabling intimate infiltration into porous cathode architectures before gelation. This in situ infiltration–solidification process can significantly improve interfacial contact with inorganic solid electrolytes, particularly sensitive sulfides and halides.

From an interfacial perspective, the ether oxygen atoms in both systems can interact with surface species of inorganic fillers. For sulfide electrolytes containing negatively charged structural units such as PS_4_^3−^ electrostatic interactions and Lewis acid–base coordination may modulate the distribution of anions (e.g., TFSI^−^), thereby altering local ion transport pathways. However, excessive interaction strength may also immobilize polymer segments, creating a trade‐off between interfacial anchoring and chain mobility.

In summary, ether‐based ring‐derived systems provide a structurally flexible platform for constructing in situ composite electrolytes. Their ultimate electrochemical performance is determined not merely by ether–Li^+^coordination but by the interplay between crystallinity modulation, polymerization kinetics, and interfacial stabilization with inorganic fillers. Cyclic ether‐derived systems should not be evaluated solely based on their intrinsic ionic conductivity. Instead, their performance in composite electrolytes emerges from a dynamic balance among crystallinity suppression, segmental motion, polymerization‐induced network formation, and interfacial chemical compatibility. Understanding these coupled structural factors is essential for rationally designing next‐generation in situ polymerized composite electrolytes.

To assist researchers in evaluating and selecting suitable CPE materials, Table 3 compiles representative examples covered in this review, detailing their structural features and performance metrics relevant to practical applications.

**TABLE 3:** Performance parameters of representative in situ polymerized composite polymer electrolytes reviewed in this article.

CPEs		Mechanism performance	Ionic conductivity (ms cm^−1^)	Electrochemical window	Cathode (vs Li^+^/Li)	Specific capacity (mAh g^−1^)	Capacity retention	References
Polymer	Inorganic filler							
PMMA	SN‐LiTFSI	Break elongation: 420%	0.49 at 30°C	4.75V	NCM811	183.50 (at 0.5 C)	82.40% after 100 cycles	[119]
PMMA/PEO/PVDF‐HFP	SiO_2_	/	8.45 × 10^−2^ at 60°C	4.70V	NCM811	148.60 (at 0.5 C); 108.40 (at 5.0 C)	81.70% after 100 cycles	[120]
PMMA/PVDF	Li_1.5_LaTe_1.5_O_6_	Elastic modulus: 1 Gpa	5.20 × 10^−3^ at 65°C	4.84V	NCM811	183.90 (at 0.2 C)	94.10% after 40 cycles	[118]
PMMA	Li_2_S	/	/	/	Li_2_S(4 mg cm^−2^)	1038 (at 0.2 C)	Reversible capacities of 471.00 mA h g^−1^ after 200 cycles)	[127]
PAN/SCN	LLZTO	/	t Li^+^ = 0.66	4.60V	LiFePO_4_	143.20 (at 0.1 C)	94.00% after 300 cycles	[154]
PAN	LLZTO/SN	/	1.90 at 25°C	4.90V	LiFePO_4_	136.20 (at 1.0 C)	90.10% after 350 cycles	[159]
PAN	LLZTO	Tensile stress: 10 MPa	2.96 × 10^−1^ at 25°C	4.80V	LiFePO_4_	149.50 (at 0.5 C); 136.60 (at 2.0 C)	96.00% after 200 cycles (at 0.5 C); 90.20% after 1000 cycles (at 2.0 C)	[157]
PAN/PEO	LLZO	/	0.29 at 25°C	4.90V	NCM811	163.80 (at 0.5 C)	90.00% after 200 cycles	[156]
PAN	Al_2_O_3_	Stress: 25 MPa with 82% strain	0.22 at 45°C	5.07V	LiFePO_4_	162.00 (at 0.2 C)	96.7% after 200 cycles	[155]
PAN	SiO_2_	Tensile strength: 2 MPa; elongation at break of 50%	8.1 at 25°C	5.10V	LiFePO_4_	102.70 (at 5.9 C)	85.10% after 580 cycles	[160]
PAN	La_2_O_3_	/	0.31 at 25°C	4.82V	LiFePO_4_	157.50 (at 0.5 C)	90.92% after 300 cycles	[161]
PAN	MnCo_2_S_4_	/	/	/	MCS@FSPAN (1C = 600 mA g^−1^)	437 (at 5.0 C)	Reversible capacities of 438.00 mA h g^−1^ after 1000 cycles)	[163]
PAN	S_7_Ge	/	/	/	S@PAN/S_7_Ge	1455.00 (at 1 A g^−1^)	77.00% after 500 cycles at 2 A g^−1^	[289]
PAN	GO/DE	/	/	2.30 V/ 2.10V	S/ACET	683.00 (at 0.2 C)	98.90% after 100 cycles	[164]
PAN/FEC	Li_6_PS_5_Cl/Li_3_N/LiF	/	/	/	LiCoO_2_	127.00 (at 0.1 C)	90.00% after 80 cycles	[167]
PEGMA/PEGDA	SiO_2_ based IBNs	The yield stress: 3.4 MPa; Tensile modulus: 338 MPa; Break elongation: 697%	6.77 × 10^−2^ at 30°C	5.14V	LiFePO_4_	140.00 (at 0.1 C)	94.28% after 40 cycles	[211]
PEGMA/HBPS	TiO_2_	/	9.00 × 10^−2^ at 30°C	/	/	/	/	[290]
PEGMA/DEPMMA	GDC	/	5.28°C at 25°C	/	/	/	/	[291]
PEGMA/PEGDME	SiO_2_	/	0.15°C at 25°C	/	/	/	/	[221]
PEGMA	PGO	Storage modulus (G′) values: 10^4^ Pa (<80°C)	0.21°C at 30°C	5.30 V (at 60°C)	V_2_O_5_(294 mAh g^−1^)	287.00 (at 0.1 C)	70.00% after 50 cycles	[215]
B‐PEGMA/PEGMEM	OV‐POSS	/	0.34°C at 25°C	5.84V	LiFePO_4_	131.10 (at 0.5 C)	92.14% after 100 cycles	[216]
PEO	LLZTO	/	/	/	LiFePO_4_	132.00 (at 2.0 C)	80.00% after 2200 cycles	[292]
PEO	OV‐LLZTO	Tensile modulus: 7.51 MPa; Compressive modulus: 20.21 MPa	0.56°C at 60°C	> 4.50V	LiFePO_4_	128.00 (at 5.0 C)	84.20% after 1000 cycles	[234]
PEO/MEMO	LLZTO	Mechanical strength: 5.65 MPa	0.216°C at 30°C	/	LiFePO_4_	146.00 (at 1.0 C)	90.90% after 200 cycles	[235]
PEO/PDA	LLZTO	Tensile strength: 2.55 MPa	0.11°C at 25°C	4.20V	LiFePO_4_	162.50 (at 0.1 C)	90.35% after 500 cycles	[236]
PEO/PVDF‐HFP	LLZO	Compression stress: 0.1 MPa	5.91 × 10^−2^ at 50°C	5.00V	LiFePO_4_	146.50 (at 0.1 C)	99.00% after 80 cycles	[239]
PEO	ZnO	/	1.50 × 10^−2^ at 25°C	4.50V	NCM811	164.70 (at 0.5 C)	82.00% after 200 cycles	[293]
PEO	SiO_2_	Young's modulus: 0.72 GPa	0.18°C at 30°C	5.30V	LiFePO_4_	161.20 (at 0.5 C)	88.00% after 400 cycles	[233]
PEO	Li_3_PS_4_	/	0.49 at 60°C	5.50V	LiFePO_4_	127.60 (at 1.0 C)	85.52% after 1000cycles	[241]
PEO	Li_2_S_6_	/	0.17°C at 40°C	/	NCM811	183.00 (at 0.03 mAh cm^−1^, cathode loading = 1.8 mg cm^−2^)	72.00% after 700 cycles	
PEO	LSPSCl	/	t Li^+^ = 0.43	/	LSPSCl‐C‐LiTFSI	414.00 (at 0.1 A g^−1^)	97.80% after 100 cycles	[242]
PEO	LiF	/	0.12°C at 50°C	/	LiFePO_4_	150.00 (at 0.1 C)	83.00% after 600 cycles	[255]
PEO	CsPbI_3_	/	1 × 10^−1^ at 30°C	5.10V	LiFePO_4_	156.00 (at 0.1 C)	93.50% after 100 cycles	[250]
PEO/PEGDME	Li_3_YBr_6_/LiNO_3_/LiTFSI	/	1°C at 25°C	4.50V	LiFePO_4_	150.20 (at 0.2 C at 60°C)	86.50% after 200 cycles	[249]
PDOL	YSZ	Stress: 160 MPa with 50% strain	0.27°C at 25°C	5.20V	NCM622	164.70 (at 0.5 C)	72.80% after 800 cycles	[269]
PDOL	YSZ/LiPF_6_	Fracture stress: 28 MPa	0.58	5.10V	LiCoO_2_	140.00 (at 0.5 C)	90.00% after 200 cycles	[273]
PDOL	TiO_2_	/	1.74 at 25°C	5.30V	LiFePO_4_	142.00 (at 1.0 C)	90.00% after 1000cycles	[39]
PDOL	LLZTO	Fracture stress:>50 MPa	0.29°C at 25°C	4.90V	NCM622	149.10 (at 0.3 C)	80.48% after 200 cycles	[268]
PDOL	LAGP	/	0.46°C at 25°C	5.30V	NCM622	147.00 (at 0.5 C)	76.00% after 400 cycles	[271]
PDOL/PVDF‐HFP	LATP	/	0.15°C at 25°C	5.30V	LiFePO_4_	138.10 (at 1.0 C)	97.72% after 300 cycles	[272]
PDOL	LiF/ LiBF_4_	/	0.19 at 30°C	4.60V	NCM811	165.30 (at 1.0 C)	84.30% after 100 cycles	[281]
PDOL	SnF_2_	/	7.2 × 10^−2^ at 45°C	/	LiFePO_4_	159.00 (at 0.1 C)	81.76% after 350 cycles	[282]
PDOL	SnCl_4_	/	t Li^+^ = 0.56	4.90V	LiFePO_4_	160.00 (at 0.2 C)	70.00% after 100 cycles	[284]
PDOL	Sn‐LiCl	/	/	/	LiFePO_4_	150.00 (at 1.0 C)	99.10% after 600 cycles	[283]
PVDF	LLZTO	Young's modulus: 30 MPa; Tensile strength: 5.92 MPa	0.50 at 25°C	/	LiCoO_2_	150.00 (at 0.4 C)	98.00% after 120 cycles	[192]
PVDF/PEO	LLZO	Tensile strength:0.88 MPa Modulus:11.43 MPa	0.10°C at 50°C	5.02V	LiFePO_4_	147.60 (at 0.2 C)	99.20 after 180 cycles	[237]
PVDF	LATP	Tensile strength: 15.3 MPa; Elongation at break of 104.4%	1.06°C at 25°C	/	LiFePO_4_	150.00 (at 0.5 C)	99.00% after 200 cycles	[191]
PVDF	LATP	/	0.60°C at 25°C	4.50V	NCM811	168.00 (at 0.1 C)	94.00% after 150 cycles	[193]
PVDF	Li_4_Ti_5_O_12_	/	0.28°C at 35°C	5.00 V	LiFePO_4_	150.00 (at 0.5 C)	99.70% after 250 cycles	[194]
PVDF‐HFP	GO/PHGB	Elongation at break 202%	/	/	LiFePO_4_	153.44 (at 0.1 C)	95.70% after 100 cycles	[188]
PVDF	LPSCl	/	1.02 at 25°C	/	/	/	/	[199]
PVDF‐HFP	LGPS	Young's modulus: 549 MPa; Elongation at break over 580%	0.18°C at 25°C	4.83V	LiFePO_4_	136.00 (at 0.2 C)	99.50 after 300 cycles	[200]
PVDF‐HFP	Li_7_PS_6_	/	0.11°C at 25°C	/	LiFePO_4_	160.00 (at 0.2 C)	98.00% after 150 cycles	[197]
PVDF/EAC	S‐CNT	/	0.70°C at 25°C	/	S‐CNT	700.00 (at 2.0 C)	90.00% after 100 cycles	[196]
PVDF‐HFP	LiCl	6.5 GPa	/	/	LiFePO_4_	139.90 (at 0.2 C)	99.10% after 130 cycles	[208]

## 5 Conclusions and Outlook

### 5.1 Performance Benefits

In light of the systematic analysis presented, the superior functional characteristics of in situ polymerized composite solid electrolytes arise from the synergistic integration of the following key elements.

#### 5.1.1 Ionic Conductivity Enhancement

The synergistic interactions between polymer chains and inorganic fillers promote the formation of continuous and smoother ion‐conduction pathways within the composite matrix. In this collaborative architecture, inorganic fillers (e.g., Li_6_PS_5_Cl discussed in Section 2.2.1.2) provide high intrinsic ionic conductivity, while polymers (e.g., PEO) facilitate increased chain segment mobility, lowering the energy barrier for ion migration.

This fundamental interplay, exemplified by the optimized organic–inorganic interfaces in the nanoparticle hybrid crosslinked system (such as mentioned in Section 2.2.1.3), yields a substantial improvement in the overall ionic conductivity of the electrolyte. Consequently, these performance enhancements, demonstrated in full‐cell configurations under practical conditions (as evidenced in Section 2.3.2.1), render the in situ polymerized CPEs highly suitable for integration into next‐generation, high‐energy‐density solid‐state lithium battery systems.

Despite the intrinsic performance advantages discussed above, ionic conductivity remains one of the primary limiting factors for composite polymer electrolytes in practical applications. While the synergistic organic–inorganic architecture provides a fundamental pathway for facilitating lithium‐ion transport, further improvements require systematic optimization across compositional design, polymerization chemistry, and interfacial transport regulation. Accordingly, potential strategies for enhancing ionic conductivity are elaborated in the subsequent outlook discussion, where hierarchical design principles and process‐level engineering considerations are systematically examined.

#### 5.1.2 Interface Compatibility Optimization

The combination of polymer chains with inorganic filler particles establishes stable chemical and physical interactions at the electrolyte‐electrode interface, which are instrumental in forming a durable SEI/CEI. Key interfacial mechanisms include the dynamic hydrogen bonding between ester groups in PMMA and oxide‐type fillers such as LLZO (as analyzed in Section 2.1.1.1), as well as coordination interactions involving –CN functional groups in PAN with sulfide particles (as discussed in Section 2.1.2.2), these molecular‐level interactions facilitate the in situ construction of a uniform and ionically conductive interphase during cycling; The incorporation of fluorine‐containing functional groups, exemplified by PVDF‐based systems, promotes the development of a robust and LiF‐rich SEI under high‐voltage conditions, this effectively suppresses detrimental side reactions, such as the formation of Li_2_CO_3_, and inhibits the growth of lithium dendrites.

This interfacial stabilization markedly improves the cycling stability and operational safety of CPEs, especially under elevated current densities and voltages, making them highly attractive for safety‐critical energy storage applications.

#### 5.1.3 Mechanical Property Modification

CPEs integrate the flexibility of polymer chains with the rigidity of inorganic fillers, yielding a “rigid‐yet‐flexible” mechanical architecture essential for solid‐state battery operation. The inorganic fillers, such as the oxides employed in PDOL‐based (Section 2.2.1) and PVDF‐based (Section 2.3.1) systems, impart substantial stiffness and enhance the overall mechanical strength, further reinforced by crosslinking within the polymer matrix. Concurrently, the inherent flexibility and long‐chain nature of the polymers effectively mitigate crack propagation and accommodate volume changes during cycling, thereby improving resistance to compressive and tensile stresses.

This synergistic reinforcement significantly prevents critical failure modes, including membrane rupture and electrode‐electrolyte delamination, which collectively contribute to exceptional long‐term cycling stability. The enhanced mechanical resilience renders CPEs particularly suitable for high‐stress energy storage applications, especially those employing lithium metal anodes.

#### 5.1.4 Chemical Stability Improvement

Compared to pure inorganic or organic electrolytes, in situ polymerized CPEs exhibit markedly improved chemical stability, particularly against ambient moisture and oxygen. This enhancement is critically attributed to the strategic incorporation of hydrophobic polymers, with fluorinated matrices such as PVDF and its copolymers playing a pivotal role (as detailed in Section 2.3.1).

In practical battery applications, this superior chemical stability ensures reliable long‐term operation even under challenging environmental conditions. For example, the exceptional moisture resistance afforded by hydrophobic polymer frameworks enables assembled cells to maintain stable cycling performance, which is a critical parameter for the viability of large‐scale, real‐world applications where controlled, dry environments cannot be guaranteed. By effectively mitigating moisture‐driven parasitic reactions, CPEs not only achieve enhanced long‐term electrochemical performance but also demonstrate improved safety and durability, positioning them as a more robust candidate for next‐generation energy storage systems.

### 5.2 Key Challenges

Notwithstanding the compelling functional merits of in situ polymerized composite solid electrolytes, their path to widespread industrial adoption and commercialization is impeded by a series of persistent hurdles in practical deployment.

#### 5.2.1 Constrained Material Portfolio and Design Flexibility

Currently, only a narrow selection of polymer–filler systems has been matured toward scalable implementation, with many compositions relying on relatively rigid and fixed pairing principles—as exemplified by the prevalent coupling of PMMA‐based matrices with sulfide‐type fillers, a representative system elaborated in Section 2.1.1.2.

This limitation originates from a fundamental lack of systematic and high‐throughput studies exploring the combinatorial space of polymer and filler chemistries. As a result, the compatibility and potential synergistic effects across a broad range of material combinations remain poorly understood, which severely restricts the ability to tailor CPE properties for specific battery requirements such as high‐energy‐density configurations, fast‐charging capability, or operation under variable environmental conditions. This narrow design space ultimately curtails their practical applicability across diverse real‐world scenarios.

#### 5.2.2 Absence of Meticulous Control in Polymerization

While in situ polymerization enables intimate interfacial contact and structural integration, it also introduces a coupled sequence of chemical and kinetic risks that extend far beyond the initial optimization of ionic conductivity. The reliability of composite polymer electrolytes is not determined solely by the final network structure, but by how the polymerization chemistry unfolds in the presence of reactive inorganic solid‐state electrolytes and how transient intermediates evolve into long‐term interfacial states.

In most in situ systems, polymerization proceeds through highly reactive radical or cationic intermediates whose lifetimes, diffusion ranges, and termination pathways are strongly influenced by local viscosity and filler dispersion. During the early stages of reaction, these transient species coexist with unreacted monomers and initiator fragments within a heterogeneous inorganic–organic environment. Surface defect sites, unsaturated bonds, or chemically labile units in sulfide or oxide electrolytes may act as unintended interaction centers. In sulfide‐based systems, localized perturbation of P–S bonding environments or subtle surface reconstruction may occur under radical exposure, whereas in oxide systems, Lewis acidic metal centers or surface hydroxyl groups can participate in side reactions with monomers or initiator‐derived species. Even when such processes are confined to nanometer‐scale interfacial regions, they alter surface chemistry and interfacial energy, thereby influencing subsequent ion transport continuity and mechanical adhesion.

As polymerization progresses, the rapid increase in crosslink density elevates viscosity and can induce vitrification before complete monomer conversion is achieved. This kinetic confinement traps residual monomers, oligomers, and initiator fragments within the polymer matrix. These species do not merely represent incomplete synthesis; they introduce chemically and mechanically heterogeneous domains. Mechanically, they reduce local modulus and generate spatial gradients in network rigidity. Chemically, they may function as redox‐active or nucleophilic centers that gradually participate in parasitic interfacial reactions during battery operation. The consequence is a system in which transient polymerization intermediates are converted into latent degradation pathways.

The presence of inorganic fillers further amplifies this coupling between reaction kinetics and structural heterogeneity. Variations in particle size distribution, morphology, and surface chemistry create non‐uniform diffusion fields and heat dissipation profiles, leading to spatially differentiated polymerization rates and crosslink densities. These reaction gradients translate into coupled gradients in ionic conductivity, mechanical strength, and electrochemical stability. Under prolonged cycling or high current densities, such heterogeneity can localize stress, promote concentration polarization, and accelerate dendritic penetration or unstable interphase evolution.

Therefore, the key challenge of in situ polymerization is not simply achieving high conductivity at the laboratory scale, but ensuring that reaction chemistry, radical lifetime, monomer conversion, and filler surface reactivity are collectively regulated to prevent interfacial perturbation and long‐term degradation. In this perspective, polymerization is not a neutral processing step but a chemically active event whose kinetics and intermediates directly shape the structural uniformity, interfacial chemistry, and durability of the composite electrolyte.

#### 5.2.3 The Black Box Phenomenon in Complex Interface

The complex interface between composite electrolytes and electrodes remains largely in a “black box” state, wherein the dynamic electrochemical and physicochemical interactions are poorly resolved at the molecular or even nanoscale level. This opacity stems from the multi‐component, spatially heterogeneous, and temporally evolving nature of the interphase region, which involves simultaneous and competing processes such as Li^+^ flux distribution, side reactions involving organic and inorganic species, and mechanical stress accumulation. Under practical operational extremes—particularly elevated temperature and stack pressure—these coupled processes become further entangled, often manifesting as resistive interphase formation, dendritic lithium nucleation, and progressive contact degradation.

A central contributor to this mechanistic ambiguity is the limited availability of operando characterization techniques capable of capturing transient processes at buried solid–solid interfaces under realistic electrochemical conditions. Conventional in situ transmission electron microscopy (TEM) and secondary ion mass spectrometry (SIMS) provide valuable structural or compositional snapshots, yet they frequently lack either sufficient temporal resolution to follow fast polymerization and interphase reactions or sufficient electrochemical representativeness to emulate full‐cell conditions.

To genuinely “open” the black box, advanced operando techniques capable of tracking kinetic evolution and phase behavior during in situ polymerization and subsequent cycling are required. in situ rheology, for example, enables real‐time monitoring of viscosity evolution and gelation dynamics, providing quantitative correlation between reaction kinetics and mechanical network formation. Such measurements are essential for understanding how precursor flow arrest influences interfacial wetting and crosslink uniformity in porous electrodes.

Similarly, operando nuclear magnetic resonance (NMR)—including time‐resolved ^1^H, ^7^Li, and diffusion‐ordered spectroscopy (DOSY) measurements—offers molecular‐level insight into polymerization kinetics, Li^+^ coordination environment evolution, and phase separation behavior. Recent developments in operando solid‐state NMR have demonstrated the capability to monitor lithium transport pathways and interfacial reactions in solid‐state batteries with chemical specificity, thereby bridging structural evolution and electrochemical signatures.

Complementary synchrotron‐based techniques such as operando X‐ray absorption spectroscopy (XAS), grazing‐incidence small‐angle X‐ray scattering (GISAXS), and time‐resolved X‐ray tomography further enable visualization of interphase growth, structural heterogeneity, and morphological evolution across multiple length scales. When coupled with electrochemical impedance spectroscopy (EIS) and mechanical stress mapping, these tools provide a multidimensional dataset linking chemical reactions, mechanical deformation, and ionic transport degradation.

The integration of these cutting‐edge diagnostics is essential for transforming interfacial optimization from empirical trial‐and‐error to mechanism‐driven design. Without time‐resolved and spatially resolved probing of polymerization kinetics, Li^+^ coordination dynamics, and phase evolution, the roles of functional groups (e.g., –C≡N in PAN discussed in Section 2.2) or filler‐induced Lewis acid–base interactions in regulating Li deposition and suppressing decomposition cannot be quantitatively engineered. Advancing operando methodologies therefore represents a prerequisite for the rational stabilization of CPEs interfaces and their reliable implementation in high‐energy‐density solid‐state batteries.

#### 5.2.4 Safety and Thermal Management Considerations

Beyond electrochemical performance, the thermal behavior of in situ polymerized CPEs during curing and operation represents a non‐trivial engineering challenge.

Most radical and ring‐opening polymerizations employed in CPEs fabrication are intrinsically exothermic, with reaction enthalpies typically ranging from 50 to 100 kJ mol^−1^ depending on monomer structure and crosslink density [294, 295]. In thin laboratory‐scale cells, this heat is rapidly dissipated. However, in thick composite cathodes (≥100 µm) [296] or multilayer pouch configurations, limited thermal conductivity of polymer matrices (∼0.2–0.4 W m^−1^ K^−1^) [297] may lead to localized heat accumulation during curing. Even a modest adiabatic temperature rise (ΔT_ad) of 10°C–30°C can significantly accelerate reaction kinetics according to Arrhenius behavior, resulting in spatially heterogeneous crosslink density.This thermal–kinetic coupling introduces several risks:

- Crosslink Gradient Formation—Elevated local temperature increases polymerization rate and crosslink density near heat‐generation zones, producing stiffness gradients across the electrolyte layer.
- Volumetric Shrinkage and Stress Accumulation—Highly crosslinked networks typically undergo curing shrinkage of 2%–8% [298]. When polymerization occurs within confined electrode pores, constrained contraction generates interfacial tensile stress, potentially exceeding several MPa depending on modulus evolution. Such stress may induce microcracks, delamination, or loss of interparticle contact.
- Thermal Runaway Coupling under Abuse Conditions—Although CPEs remove flammable liquid solvents, exothermic polymer decomposition (commonly initiated above 250°C–400°C) can contribute additional heat during thermal abuse [299, 300] If interfacial degradation has already created resistive hotspots, localized Joule heating may synergistically amplify temperature rise.

At present, most studies evaluate curing behavior using differential scanning calorimetry (DSC) under idealized conditions, whereas in situ temperature evolution inside full cells is rarely monitored. Quantitative mapping between polymerization heat release, cell geometry, and resulting thermal gradients remains largely unexplored.

Collectively, these observations indicate that thermal behavior during in situ polymerization is not merely a safety concern but a coupled thermo–kinetic–mechanical problem. The interplay among reaction enthalpy, heat diffusion, crosslink evolution, and geometrical confinement governs the spatial uniformity of the electrolyte network and ultimately determines mechanical reliability and electrochemical stability at practical cell scales. Importantly, as device thickness, areal capacity, and manufacturing complexity increase, this coupling does not diminish but instead becomes further intertwined with precursor transport, rheological evolution, and architectural heterogeneity. Addressing thermal regulation at laboratory scale is therefore only the first step; true scalability demands a broader understanding of how reaction kinetics and network formation interact with flow behavior and structural complexity in realistic battery configurations.

#### 5.2.5 Scalability and Engineering Bottlenecks for Practical Cell Implementation

If Section 4.2.4 highlights thermo–kinetic–mechanical coupling during curing, scalability introduces an equally critical yet distinct constraint: a rheological–kinetic–mechanical–architectural coupling that emerges when in situ polymerization is translated from thin laboratory cells to high‐energy‐density battery formats.

In practical architectures, electrolytes must be formed within thick composite cathodes (≥100 µm) supporting areal capacities of 3–5 mAh cm^−2^or higher. Under these geometrical conditions, precursor transport is governed by time‐dependent rheology that is intrinsically dictated by reaction kinetics. As polymerization proceeds, viscosity increases nonlinearly until gelation arrests flow. The competition between infiltration depth and curing rate therefore determines whether the electrolyte can fully wet and penetrate the porous electrode scaffold before network solidification occurs.

This dynamic evolution directly couples to mechanical development. As the polymer network forms within confined pores, modulus rises while stress relaxation capability diminishes. Any spatial heterogeneity in curing—originating from kinetic gradients or incomplete infiltration—translates into mechanically discontinuous regions. Under practical stack pressures and cycling‐induced volume fluctuations, such heterogeneity can amplify stress concentration and promote interfacial instability.

Architectural scaling further intensifies these sensitivities. Large‐area pouch‐cell formats demand compositional homogeneity, uniform filler dispersion, and consistent curing across extended geometries. Minor variations in precursor formulation or environmental conditions during continuous processing may propagate into conductivity gradients and crosslink‐density variations at the device scale.

Scalability is therefore governed by the coupled interplay of rheological evolution, reaction kinetics, mechanical accommodation, and architectural complexity. The engineering bottleneck is not merely intrinsic material performance, but the ability to establish a spatially uniform, mechanically robust, and electrochemically continuous electrolyte network under realistic areal loading and manufacturing constraints.

### 5.3 Strategies and Future Perspectives

In response to these barriers, we formulate a systematic roadmap of targeted strategies and future priorities, designed to overcome the key impediments to the scalable deployment and commercialization of in situ polymerized CPEs.

#### 5.3.1 Data‐Driven Material Design for System Expansion

The compositional design space of in situ polymerized composite electrolytes extends far beyond the selection of individual materials. Practical CPEs systems typically consist of multiple interdependent components—including polymerizable monomers, lithium salts, inorganic fillers, crosslinkers, plasticizers, and interfacial modifiers—whose synergistic interactions collectively determine ionic conductivity, mechanical integrity, and interfacial stability. The high‐dimensional coupling among composition, stoichiometry, and processing parameters renders empirical optimization increasingly inefficient and costly.

To address this complexity, machine learning (ML) offers a powerful framework for navigating multidimensional formulation spaces rather than merely screening isolated materials. In multi‐component systems, ML‐driven optimization can simultaneously account for compositional ratios, crosslink density, filler loading levels, and processing conditions, enabling the identification of globally optimized combinations under multiple performance constraints. From the perspective of ionic conductivity, ML‐enabled multi‐objective optimization is particularly valuable for identifying high‐σ formulations that simultaneously maximize salt dissociation, maintain sufficient segmental mobility, and construct percolated inorganic–organic transport networks without excessive viscosity or phase separation.

The implementation of ML‐driven discovery follows a structured workflow encompassing: (1) construction of curated material databases incorporating structural descriptors (e.g., lattice energy, bandgap, ionic radius), thermodynamic properties, and synthetic parameters; (2) feature engineering to identify critical correlations between intrinsic material characteristics and functional performance; (3) training of ensemble models capable of predicting key properties with validated accuracy. In a seminal demonstration, Chen et al. implemented a hierarchical screening platform that processed 20 717 lithium‐containing compounds through successive filtering stages. Their approach integrated random forest classifiers for initial phase stability assessment with gradient boosting regression for ionic conductivity prediction, achieving a >90% accuracy in identifying superionic conductors. This data‐driven pipeline, validated by molecular dynamics simulations, identified Li_3_BiS_3_as a promising filler material exhibiting exceptional room‐temperature conductivity (>10 mS cm^−1^) and compatible interfacial characteristics with polyether matrices. The versatility of ML frameworks enables tailored solutions for specific CPEs design challenges. For example, for sulfide‐based solid electrolytes, support vector regression models trained on structure‐activity relationships have successfully predicted ionic migration barriers with mean absolute errors <0.1 eV. In polymer‐ceramic composite systems, graph neural networks operating on molecular graph representations have screened 329 candidate pairs for lithium‐metal compatibility, identifying PAN‐LLZO composites with 40% improved cycling stability compared to conventional formulations. These data‐driven approaches not only accelerate the discovery of novel compositions but also establish quantitative structure‐property relationships that fundamentally advance our understanding of ion transport mechanisms in heterogeneous electrolyte systems.

Importantly, multi‐component ML frameworks enable multi‐objective optimization, where trade‐offs between ionic mobility, mechanical strength, electrochemical window, and processing feasibility can be quantitatively balanced. Such approaches are particularly relevant for in situ polymerized systems, in which polymerization kinetics, crosslink density, and filler dispersion must be co‐optimized to mitigate the intrinsic design contradictions discussed earlier.

While significant progress has been made, current ML methodologies face limitations in predicting long‐term interfacial evolution and accounting for non‐equilibrium processing effects. Future developments should incorporate time‐dependent descriptors, reaction kinetics parameters, and operando characterization data into training datasets. The integration of Bayesian optimization with autonomous synthesis platforms offers a promising pathway toward closed‐loop formulation optimization, enabling rapid convergence on high‐performance multi‐component CPEs systems.

#### 5.3.2 Precision Control of Polymerization via Advanced Synthetic Techniques

The inherent challenges in regulating in situ polymerization—often resulting in residual reactive monomers and oligomers—pose a significant barrier to achieving ideal interfacial compatibility in composite polymer electrolytes. In response to this challenge, controlled polymerization techniques have emerged as a foundational strategy for precise manipulation of macromolecular structure and conversion efficiency.

Reversible addition‐fragmentation chain‐transfer (RAFT) polymerization stands as one of the most effective methods, operating through a reversible chain‐transfer mechanism that affords exceptional control over polymer architecture and narrow molecular weight distribution. Its utility has been demonstrated in the synthesis of well‐defined block copolymers for solid polymer electrolytes, significantly reducing residual monomer content and improving mechanical‐ionic balance. Similarly, atom transfer radical polymerization (ATRP) and nitroxide‐mediated polymerization (NMP) offer robust pathways for tailoring polymer topology and terminal functionality, enabling the fabrication of electrolytes with highly uniform network structures. Beyond these established methods, emerging systems such as organotellurium‐mediated polymerization (TERP) and organostibine‐mediated polymerization (SBRP) exhibit distinctive strengths in the controlled polymerization of non‐conjugated monomers. Furthermore, the integration of living polymerization with click‐chemistry approaches has enabled the construction of multidimensional polymer architectures with enhanced functionality and interfacial adaptability. To be mentioned, conductivity improvement should be considered a direct output of polymerization‐pathway engineering: controlled polymerization can suppress over‐crosslinking and compositional micro‐heterogeneity, thereby preserving segmental dynamics and reducing ion‐trapping domains. By minimizing residual monomers/oligomers while maintaining a mechanically robust yet mobile network, these approaches can increase σ and lower activation barriers for Li^+^ transport without sacrificing interfacial compatibility.

The focus must shift from traditional empirical methods to the deliberate engineering of polymerization pathways, where reaction kinetics, radical control, and monomer interaction with inorganic fillers are strategically designed. This shift will enable precise modulation of interfacial interactions, ensuring chemical stability, optimizing ionic conductivity, and enhancing long‐term electrochemical performance, ultimately creating robust and durable composite electrolytes for next‐generation solid‐state batteries.

Looking forward, the fusion of controlled polymerization with intelligent process control represents the next evolutionary step in electrolyte engineering. Deep learning models are increasingly capable of predicting optimal initiator ratios and reaction trajectories based on historical data, enabling pre‐emptive tuning of polymerization pathways. The ultimate vision is a closed‐loop autonomous synthesis system, wherein real‐time data from in situ spectroscopic sensors dynamically guide reaction conditions—fine‐tuning temperature, pressure, and reagent feed rates to ensure reproducible and high‐fidelity CPEs fabrication. Such an integrated approach, rooted in precision polymer chemistry and augmented by adaptive control systems, is essential to bridge the gap between laboratory‐scale innovation and industrially viable production of next‐generation solid‐state batteries.

#### 5.3.3 Illuminating the Interfacial ‘Black Box’ Through Multi‐Modal Analysis

The complex and dynamically evolving nature of interfacial reactions within composite solid‐state electrolytes presents a fundamental challenge for direct observation and analysis. Conventional ex‐situ methods fail to capture the transient processes and dynamic evolution at buried interfaces, resulting in limited understanding of the underlying mechanisms that govern interfacial stability and degradation.

In situ characterization techniques have revolutionized our ability to probe these complex interfacial phenomena under operational conditions. A particularly powerful approach involves the multi‐modal integration of complementary in situ techniques, which enables simultaneous tracking of structural, chemical, and morphological evolution across different length and time scales. For instance, while in situ transmission electron microscopy (TEM) provides exceptional spatial resolution for visualizing structural changes at the nanoscale, it can be effectively correlated with in situ X‐ray photoelectron spectroscopy (XPS) to simultaneously monitor corresponding chemical state evolution at the interface. This combined approach was exemplified in the work of Cretu et al., where a specially designed LFP|LAGP|LVP micro‐solid‐state battery within TEM revealed how mechanical stress from grain boundaries combines with Li^+^ diffusion gradients to initiate interfacial failure mechanisms.

Beyond structural‐chemical correlations, the integration of vibrational spectroscopy techniques such as in situ Raman spectroscopy offers additional dimension to interface analysis, enabling real‐time tracking of molecular configuration changes and phase transitions during electrochemical cycling. Similarly, specialized techniques including in situ liquid secondary ion mass spectrometry (SIMS) have demonstrated unique capabilities for probing ionic transport and compositional gradients across interfaces, as evidenced by Yu et al.’s quantitative mapping of Li^+^ transport pathways within the solid‐electrolyte interphase.

Notably, the conductivity relevant to practical performance is often an effective transport metric jointly determined by bulk σ and interfacial continuity. Operando‐resolved descriptors—such as interphase growth rate, local Li^+^ coordination evolution, and stress‐assisted contact loss—can therefore guide interface designs that suppress space‐charge accumulation and reduce interfacial impedance. Such mechanistic‐to‐quantitative linkage is essential to translate intrinsically conductive CPEs chemistries into consistently low‐resistance, continuous Li^+^ pathways under practical current densities.

The progressive deployment of advanced operando diagnostics does more than merely illuminate previously hidden interfacial processes—it fundamentally transforms qualitative observations into quantifiable descriptors. Time‐resolved measurements of polymerization kinetics, Li^+^ coordination environments, phase separation dynamics, and stress evolution generate multidimensional datasets that capture the spatiotemporal complexity of composite electrolyte interfaces. Once these transient variables are parameterized—such as diffusion coefficients, reaction rate constants, modulus evolution, or interphase growth rates—they become amenable to statistical correlation and predictive modeling. In this context, the transition from a “black box” paradigm to a data‐driven framework represents a conceptual shift from post hoc interpretation toward predictive interface engineering. Rather than empirically adjusting functional groups or filler composition, interfacial stability can be optimized through models trained on experimentally derived kinetic, thermodynamic, and mechanical descriptors. The integration of operando characterization with machine learning thus establishes a closed‐loop strategy, wherein mechanistic insight informs data generation, and data‐driven models guide rational material and interface design.

The future of interfacial analysis lies in advancing these multi‐modal platforms toward higher temporal resolution, improved spatial correlation, and more realistic electrochemical environments. By developing integrated systems that simultaneously combine multiple characterization modalities, researchers can overcome the limitations of individual techniques and construct a comprehensive picture of interfacial phenomena. This experimental framework, complemented by targeted computational insights, is poised to transform our understanding of solid‐state interfaces from phenomenological description to mechanistic prediction, ultimately enabling the rational design of stable interphases for next‐generation batteries.

#### 5.3.4 Reaction Engineering and Thermal Regulation

In direct response to the thermo–kinetic–mechanical coupling identified in Section 4.2.4, mitigation strategies must regulate not only reaction enthalpy but the dynamic interplay among heat generation, reaction kinetics, and network evolution. Thermal management during in situ polymerization cannot be treated as an isolated safety parameter; it requires an integrated reaction‐engineering framework that quantitatively links polymerization thermodynamics, heat diffusion, crosslink development, and mechanical stress accumulation across relevant length scales. By explicitly coordinating these variables, reaction engineering transforms curing from an empirically controlled step into a predictively managed process, thereby stabilizing spatial uniformity and mechanical integrity under realistic device geometries.

At the thermochemical level, reducing the intrinsic enthalpy of polymerization directly lowers the potential adiabatic temperature rise, thereby suppressing the amplification of reaction rate via Arrhenius acceleration. Rational monomer design, moderated reactive group density, and selection of lower‐exotherm reaction pathways collectively constrain volumetric heat release and stabilize spatial temperature profiles in thick electrodes.

Kinetic regulation further ensures that heat generation rate does not exceed local heat diffusion capacity. By distributing network formation temporally—through staged curing or controlled initiation intensity—the peak exothermic rate can be flattened without compromising final conversion. Such regulation minimizes crosslink density gradients and mitigates stiffness heterogeneity that otherwise arises from localized thermal acceleration.

Concurrently, enhancing thermal transport within the composite electrolyte improves heat dissipation across confined geometries. Increasing effective thermal conductivity reduces temperature gradients that drive non‐uniform modulus evolution and shrinkage‐induced stress. When reaction uniformity is preserved, interfacial tensile stress and microcrack formation within electrode pores can be substantially suppressed.

These mitigation strategies must be evaluated within realistic geometrical constraints. Coupled heat‐transfer and reaction‐kinetics simulations enable quantitative prediction of temperature evolution as a function of electrode thickness, stacking configuration, and boundary heat flux. By comparing reaction rate to heat diffusion capacity, such models provide a scalable criterion for determining thermally stable curing regimes in large‐format cells.

Finally, translating these predictive frameworks into practical validation requires operando thermal diagnostics within full‐cell architectures. Real‐time temperature mapping during curing and abuse testing establishes direct correlations between reaction thermodynamics, interfacial integrity, and safety performance. Through this integration of thermochemical moderation, kinetic regulation, transport engineering, and geometrical modeling, in situ polymerization can evolve from an empirically optimized process into a predictively engineered manufacturing step compatible with industrial‐scale solid‐state batteries.

#### 5.3.5 Cost‐Driven Material Selection and Streamlined Process Engineering

If Section 4.2.5 reveals scalability as a rheological–kinetic–mechanical–architectural coupling problem, its resolution requires an equally integrated manufacturing‐oriented framework. Practical implementation demands synchronized control over precursor flow behavior, curing kinetics, mechanical evolution, and structural uniformity within high‐areal‐capacity and large‐format cells.

Rather than optimizing materials solely for peak electrochemical metrics, process‐compatible design must align rheological stability with infiltration depth, balance crosslink density with stress relaxation capability, and ensure compositional homogeneity under continuous production conditions. Only by integrating material design with manufacturing architecture can laboratory‐scale performance be translated into reproducible pouch‐cell fabrication. Current research addresses these challenges through parallel advancements in material economy and process efficiency. The systematic identification of cost‐effective materials, including ether‐based monomers and established polymer matrices like PVDF and PAN, provides a foundation for reducing raw material expenses while preserving electrochemical functionality. Simultaneously, process intensification through energy‐efficient initiation methods such as low‐energy UV curing and simplified one‐pot synthesis routes significantly enhances production efficiency. These integrated approaches effectively mitigate filler agglomeration while eliminating complex multi‐step procedures, establishing a robust framework for reproducible CPEs manufacturing.

Collectively, the transition from laboratory optimization to industrial viability requires the transformation of in situ polymerization from a materials‐driven concept into a reaction‐ and process‐engineered manufacturing strategy.

Future CPEs formulations should be explicitly tailored for compatibility with high‐throughput production systems, particularly roll‐to‐roll processing, demanding precise optimization of rheological properties and curing kinetics. Additionally, incorporating comprehensive life‐cycle assessment during early development phases becomes essential for evaluating environmental impact and recyclability. The establishment of this multidimensional alignment—spanning electrochemical performance, production economics, and sustainability—represents the decisive step toward commercial adoption in cost‐driven applications including consumer electronics and electric vehicles.

### 5.4 Summary and Outlook

In situ CPEs represent a scalable and promising platform for enabling safe, high‐energy‐density solid‐state lithium batteries. This review systematically analyzes the design of diverse polymer matrices and inorganic fillers, highlighting the critical role of organic–inorganic interfacial interactions in enhancing ionic conductivity, mechanical strength, interfacial compatibility, and chemical stability.

While substantial progress has been achieved, four critical challenges continue to impede practical implementation: (1) constrained material design space and limited compositional flexibility; (2) inadequate control over polymerization kinetics and structural uniformity; (3) unresolved complex interfacial reaction mechanisms; and (4) significant scalability limitations in manufacturing. To address these bottlenecks, innovative strategies are being developed, including machine learning‐accelerated material discovery, precision polymerization techniques, advanced in situ characterization coupled with theoretical modeling, and economically viable manufacturing protocols.

**FIGURE 16:**
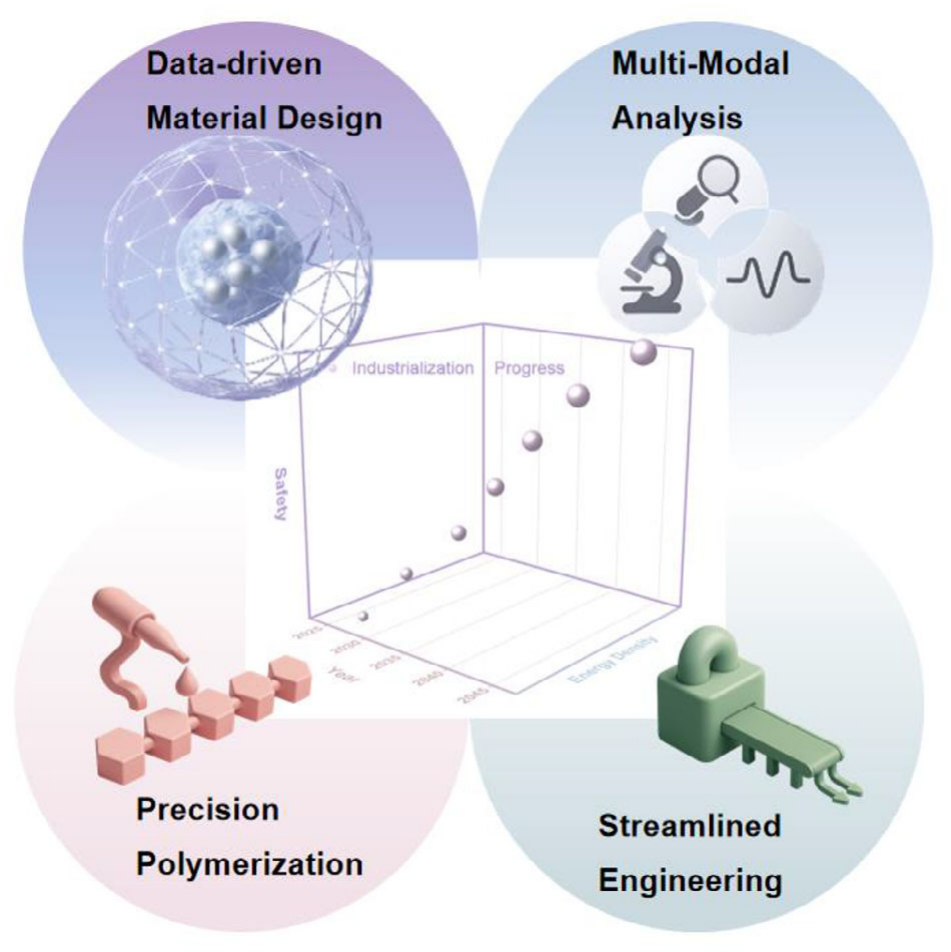
Challenges and Roadmap for in situ Polymerized Composite Solid Electrolytes.

The pathway toward commercialization necessitates deeper integration of data‐driven design methodologies with fundamental mechanistic understanding and scalable engineering solutions (Figure 16). This synergistic approach will not only bridge existing knowledge gaps but also accelerate the transition from laboratory innovation to industrial implementation. Collectively, the roadmap outlined in this review is intended to serve as a conceptual reference for guiding scientific exploration rather than as a prescriptive constraint on material selection or performance realization. With continued interdisciplinary efforts across these research domains, the widespread deployment and commercial adoption of in situ polymerized CPEs are anticipated to materialize within the next two decades, ultimately enabling a new generation of energy storage systems.

## Conflicts of Interest

The authors declare no conflicts of interest.

## Data Availability

The data that support the findings of this study are available from the corresponding author upon reasonable request.
